# Runs of homozygosity and distribution of functional variants in the cattle genome

**DOI:** 10.1186/s12864-015-1715-x

**Published:** 2015-07-22

**Authors:** Qianqian Zhang, Bernt Guldbrandtsen, Mirte Bosse, Mogens S Lund, Goutam Sahana

**Affiliations:** Department of Molecular Biology and Genetics, Center for Quantitative Genetics and Genomics, Aarhus University, Tjele, DK-8830 Denmark; Animal Breeding and Genomics Centre, Wageningen UR Livestock Research, Wageningen, 6700 AH The Netherlands

**Keywords:** Runs of homozygosity, Polymorphisms, Inbreeding, Cattle, Genome sequencing

## Abstract

**Background:**

Recent developments in sequencing technology have facilitated widespread investigations of genomic variants, including continuous stretches of homozygous genomic regions. For cattle, a large proportion of these runs of homozygosity (ROH) are likely the result of inbreeding due to the accumulation of elite alleles from long-term selective breeding programs. In the present study, ROH were characterized in four cattle breeds with whole genome sequence data and the distribution of predicted functional variants was detected in ROH regions and across different ROH length classes.

**Results:**

On average, 19.5 % of the genome was located in ROH across four cattle breeds. There were an average of 715.5 ROH per genome with an average size of ~750 kbp, ranging from 10 (minimum size considered) to 49,290 kbp. There was a significant correlation between shared short ROH regions and regions putatively under selection (*p* < 0.001). By investigating the relationship between ROH and the predicted deleterious and non-deleterious variants, we gained insight into the distribution of functional variation in inbred (ROH) regions. Predicted deleterious variants were more enriched in ROH regions than predicted non-deleterious variants, which is consistent with observations in the human genome. We also found that increased enrichment of deleterious variants was significantly higher in short (<100 kbp) and medium (0.1 to 3 Mbp) ROH regions compared with long (>3 Mbp) ROH regions (*P* < 0.001), which is different than what has been observed in the human genome.

**Conclusions:**

This study illustrates the distribution of ROH and functional variants within ROH in cattle populations. These patterns are different from those in the human genome but consistent with the natural history of cattle populations, which is confirmed by the significant correlation between shared short ROH regions and regions putatively under selection. These findings contribute to understanding the effects of inbreeding and probably selection in shaping the distribution of functional variants in the cattle genome.

**Electronic supplementary material:**

The online version of this article (doi:10.1186/s12864-015-1715-x) contains supplementary material, which is available to authorized users.

## Background

Dairy cattle have been subjected to more than 60 years of intense selection for traits to enhance milk production [1–4]. Relatively few bulls were chosen to produce thousands of daughters, resulting in large half-sib families. Traditionally, cattle breeders estimate the inbreeding coefficient by the degree of parental relatedness using pedigree and genotype data [5–8]. The rate of inbreeding in cattle populations has increased in recent years [6, 9, 10], and there is a strong correlation between inbreeding levels and reduced fitness [11]. This can be explained by the increased risk of homozygosity for deleterious alleles as inbreeding increases [12]. Thus, high levels of inbreeding in populations will result in inbreeding depression [10, 13]. Reduced variability also leads to a reduced selection response in breeding programs [14]. Thus, maintaining genetic diversity is crucial in cattle breeding populations.

The availability of high-throughput whole genome sequencing for substantial numbers of animals has opened new avenues in examining genetic diversity and led to reliable and detailed investigation of large chromosome segments, including stretches of homozygous genomic regions [15]. Runs of homozygosity (ROH) are contiguous homozygous stretches in an individual genome due to transmission of identical haplotypes from parents to offspring. ROH detection can be used to improve mating systems and minimize inbreeding. However, the effects of inbreeding vary among individuals and populations, and it has long been of interest to explore the mechanisms of inbreeding depression and deleterious variants at the genomic level [16–18]. Recently, ROH extracted from SNP chip data have been used to study the population history of different cattle breeds [19]. Purfield *et al.* [19] claimed that both natural and artificial selection of cattle, as well as demographic processes, have resulted in breeds with extensive phenotype variation. ROH may provide useful information on how these processes work in disparate populations, especially for cattle due to recent intense selection of sires, artificial insemination, and embryo transfer in some cattle breeds [19]. However, ROH estimated from SNP chip data may miss short and medium ROH due to limited resolution. Additionally, SNP arrays suffer from ascertainment biases due to inclusion of SNPs with high minor allele frequencies [20].

Consanguineous mating, population size reduction, and selection result in long homozygous regions along the genome [15, 16]. Two copies of a genome segment in an individual inherited from a common ancestor without recombination are identical-by-descent (IBD). ROH that arise due to inbreeding tend to be fairly long and are dispersed more or less randomly throughout the genome. Accumulation of deleterious variants by definition creates fitness consequences, particularly when homozygous [20, 21]. Distribution of functional homozygote variants provides information on enrichment of deleterious homozygotes within homozygous regions compared with neutral homozygotes. Charlesworth *et al.* [22] suggested that a large number of weakly deleterious variants are purged by negative selection. A human genome study [23] indicated that purifying selection interacted with founder effects during demographic processes, affecting the proportion of recessive deleterious variants. Recently, based on variant annotations, Szpiech *et al.* [24] reported long ROH enriched deleterious variation in the human genome. By counting deleterious and neutral variants inside and outside ROH in humans, they determined that out of the two competing hypotheses regarding patterns of deleterious variation in the human genome — namely, whether a smaller or larger proportion of all deleterious homozygotes resided in ROH regions compared with neutral homozygotes [24], a larger proportion of deleterious homozygote variants were found, and deleterious variants in long ROH accumulated more rapidly compared to short and medium ROH in the human genome.

Cattle domestication was initiated approximately 10,000 years ago, with evidence of at least two separate domestication events [25]. During the last few decades, breeders in modern dairy cattle breeding programs have implemented strong artificial selection. Kim *et al.* showed that selection increases overall autozygosity across the genome, whereas the autozygosity in an unselected line does not change significantly across most chromosomes in cattle populations [26]. Genomic regions under positive selection show increased ROH levels due to local reductions in haplotype diversity, *i.e.* selective sweeps [27]. Certain haplotypes under these conditions constitute a large proportion of the total haplotype pool for the portions of the genome targeted for selective sweeps. In these conditions, a portion of the ROH is the actual selection target and is retained in the ROH region [28]. These ROH will increase in frequency and be subject to purging, as the spread of haplotypes carrying deleterious recessive alleles are restrained by forces counteracting the selection pressure driving the selective sweep [29]. Therefore, we expect that this ROH type would be on average shorter, composed of more common haplotypes, be concentrated in fewer genomic locations, and contain fewer deleterious alleles than the genomic average.

Short ROH are generally due to older haplotype relatedness, while longer ROH result from more recent parental relatedness [30]. Thus, short and medium sized ROH have been subject to selection for a longer period of time than longer ROH. Furthermore, recombination will have had more time to trim down ROH that have been the target of selective sweeps [26, 31]. ROH regions are expected to exhibit an increased frequency of homozygotes compared to non-ROH regions, as the homozygote allelic frequency in non-ROH regions is p^2^ and the allelic frequency in ROH regions is p, given a population allelic frequency of p. Therefore, IBD results in enrichment of homozygotes in ROH. Given the nature of cattle breeding, artificial selection is expected to play a more crucial role in shaping the frequency and distribution of functional variants in ROH in modern cattle relative to human populations.

Therefore, we expect that selection pressures could reduce the number of regions with an increased frequency of deleterious variations, and, at the same time, enrich for short ROH regions with substantial beneficial effects in cattle breeding populations. This remains an ongoing process, as the properties of genomic variants within ROH regions continue to be discovered in cattle [32–34]. An increase in functional variant frequency in different ROH length classes should also be examined under different population-genetic processes, such as the number of generations of inbreeding [30].

Genome scale bioinformatics annotations are available from a number of sources, including the Variants Effect Predictor from ENSEMBL [35], with several available levels of annotation. Generally, synonymous polymorphisms are the least subject to selective forces as they have the least effect on the resultant protein. Tools such as SIFT can be used to predict non-synonymous change effects on proteins, and, thus, give an idea of their likely selective pressure [36]. SIFT classifies non-synonymous changes as either non-deleterious or deleterious, based on the predicted effect on the protein. By comparing these classes (synonymous, non-deleterious non-synonymous, and deleterious non-synonymous), we can study how selection has shaped deleterious variants within the cattle genomic ROH.

We hypothesize that due to strong artificial selection pressures and demographic processes in cattle, deleterious variants increased in frequency within ROH compared with non-deleterious variants and that the deleterious allelic frequency and distribution in ROH classes differs markedly from the human genome. Testing this hypothesis will contribute to our understanding of inbreeding in cattle and help elucidate how artificial selection and other population level processes affect the distribution of functional variants. To accomplish this, we examined patterns of ROH detected using full genome sequencing in four Danish cattle breeds and studied the distribution and frequency of deleterious and non-deleterious variations in different length ROH regions.

## Results

### General statistics

**Fig. 1:**
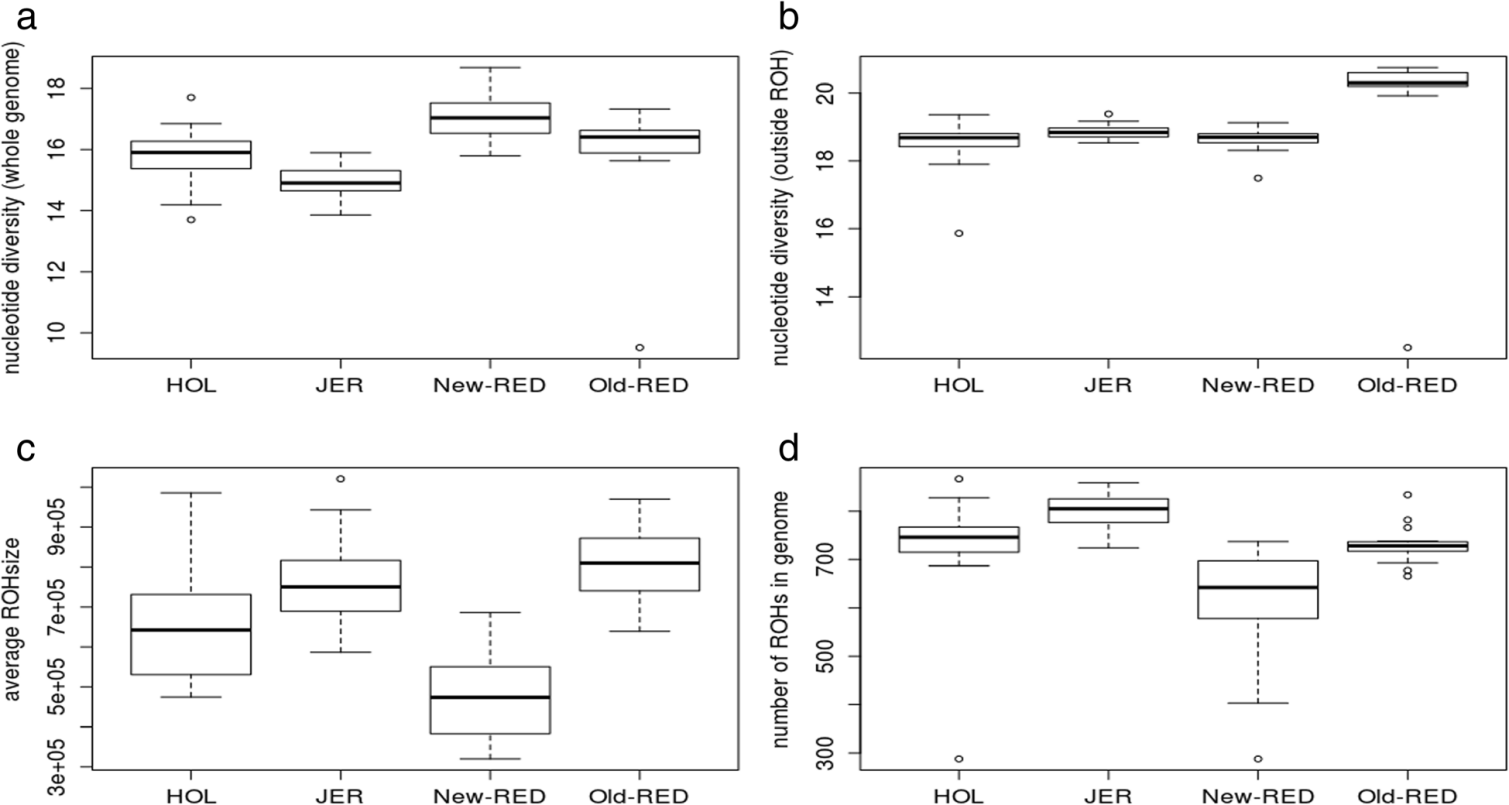
ROH general statistics. **a** Average genome-wide nucleotide diversity (polymorphic sites per 10,000 bp); **b** Average nucleotide diversity outside ROH (polymorphic sites per 10,000 bp); **c** Average ROH size (bp); **d** Average genome-wide ROH totals

Runs of homozygosity (ROH) in the autosomes of 104 resequenced individuals were determined from four Danish dairy cattle breeds: Holstein (HOL), Jersey (JER), old Red Danish Dairy cattle (old-RED), and New Danish Red Dairy cattle (new-RED) (Fig. 1 and Additional file 1: Figure S1). The average genomic ROH content was 19.5 % across the four cattle breeds, with HOL, JER, New-RED, and Old-RED having 18.67 %, 24.23 %, 11.84 %, and 23.26 %, respectively. The average number of ROH per genome was 715.5 ± 21.0, with an average size of 750,564.2 bp, ranging from 10 kbp (the minimum size considered) to 49,290 kbp (Additional file 10: Table S1). The mean ROH size varied significantly between HOL, JER, Old-RED, and New-RED (*P* < 0.001) with the exception of JER and Old-RED (Fig. 1c). The mean number of ROH was significantly different between HOL, JER, Old-RED, and New-RED cattle (*P* < 0.001) with the exception of HOL and Old-RED (Fig. 1d).

The average genome-wide nucleotide diversity (π) was 1.59 (± 0.024) heterozygous positions per kbp across all individuals, including ROH, and 1.89 (± 0.018) heterozygous positions per kbp across all individuals in the genome excluding ROH (π-out) (Additional file 10: Table S1). The minimum and maximum nucleotide diversities were 1.50 (± 0.029) SNPs/kbp in JER and 1.71 (±0.024) SNPs/kbp in New-RED, respectively (Fig. 1a and Additional file 2: Figure S2, Additional file 10: Table S1) and were significantly different between all cattle breeds except HOL and New-RED (*P* < 0.05). π-out was significantly different between Old-RED and HOL, JER, and New-RED (*P* < 0.001, two-tailed *t*-test) (Fig. 1a and b). Nucleotide diversity was higher in the vicinity of the major histocompatibility complex (MHC) on chromosome 23 (Additional file 1: Figure S1).

### Genomic patterns of homozygosity

**Fig 2:**
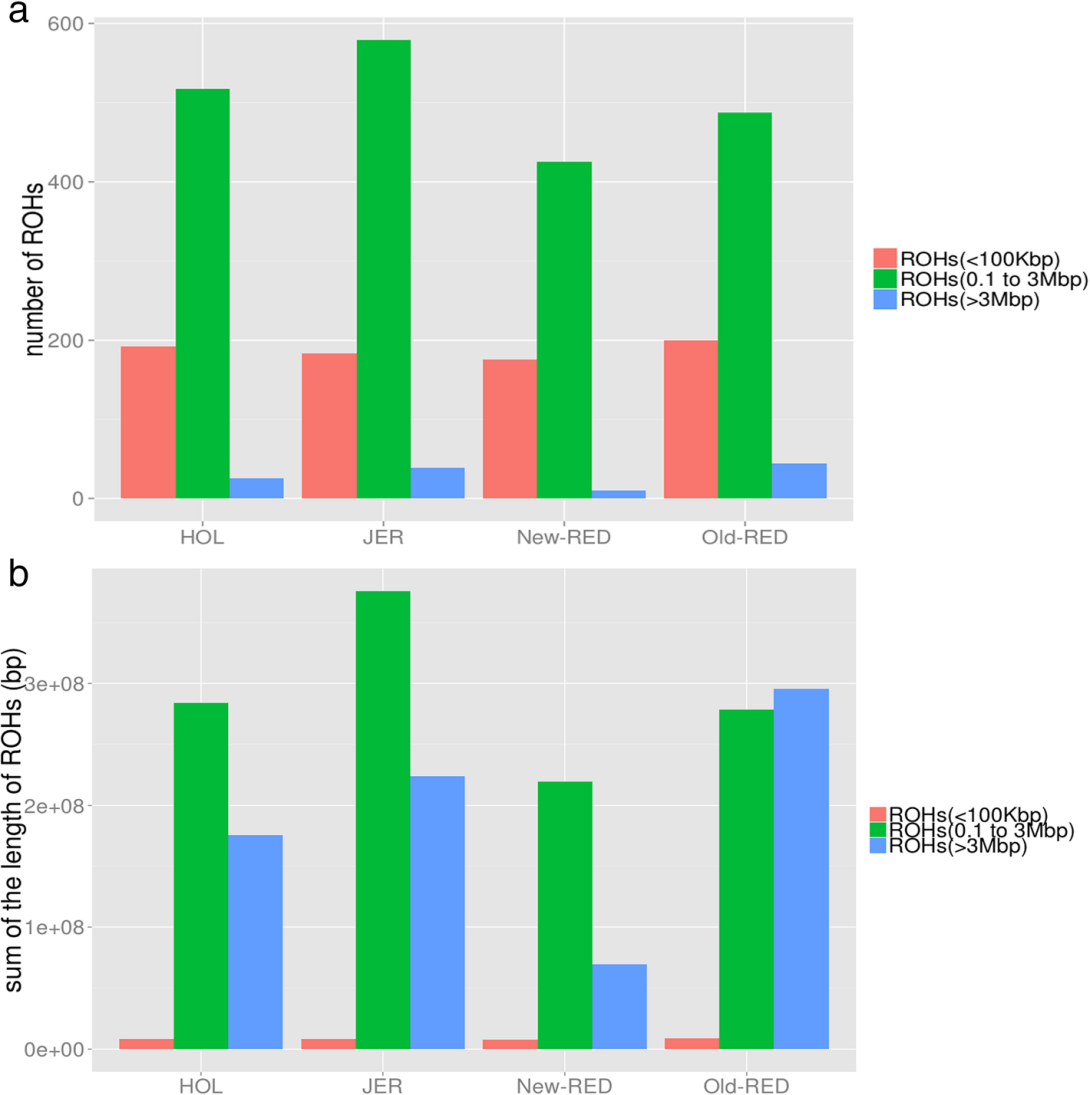
Total ROH number and genome proportions. **a** The average small (< 100 kbp, Red), medium (0.1 to 3 Mbp, Green), and large (> 3 Mbp, Blue) ROH numbers for the four breeds; **b** Average total genome ROH coverage for a given size class within each breed

ROH were separated into three size classes: small (10 kbp to 100 kbp), medium (0.1 to 3 Mbp), and large (> 3 Mbp) (as described in the Materials and Methods section). The proportion of ROH in each size class was computed in all 104 sequenced individuals. While small ROH were frequent throughout the genome, they constituted a small proportion of the entire genome (Fig. 2). In contrast, medium ROH were much less frequent, they constituted significantly more of the genome than either small or large ROH. Large ROH were at least tenfold less numerous than medium ROH, but nevertheless covered a sizable proportion of the total genome length. Old-RED cattle on average had the largest proportion of their genome in large ROH. New-RED cattle had fewer genomic ROH and a smaller genomic proportion of ROH than HOL and JER cattle (*P* < 0.001). Old-RED and JER cattle on average had more ROH and increased proportion of genomic homozygosity.

Principal component analysis successfully differentiated the four cattle breed individuals into different clusters based on SSR sequence data (Additional file 4: Figure S4), with JER the most distantly related based on PCA results. Three-dimensional plots did not show clear separation of cattle breeds based on to ROH size, ROH number, and π-out (Additional file 5: Figure S5). New-RED cattle represented the most variable cluster due to high nucleotide diversity and fewer ROH (P < 0.001) (Fig. 1). There was lower nucleotide diversity and more total ROH in JER compared to HOL and New-RED (P < 0.001) (Fig. 1). Despite its most distant origin, JER had lower nucleotide diversity (Additional file 4: Figure S4 and Additional file 5: Figure S5), creating several clusters in the three dimensional plot (Additional file 5: Figure S5). However, all Danish cattle breeds were more or less clustered together, with the exception of two New-RED individuals (Additional file 5: Figure S5).

Based on sequence data, New-RED cattle exhibited the fewest ROH and smallest ROH sizes. This is a composite breed with contributions from other red breeds, including Swedish Red, Finnish Ayrshire, and Brown Swiss. Compared with New-RED, these data suggest that the Old-RED breed has been more inbred based on relatively high coverage of long regions of homozygosity (> 3 Mbp) (Additional file 5: Figure S5), probably due to a relatively small breeding population and recent years of close mating.

Furthermore, the sharing of ROH regions was examined among sequenced individuals (Additional file 15: Figure S10B and Additional file 16: Figure S11C). Sharing of ROH regions primarily happened in short rather than long ROH regions, likely a result of combination of inbreeding and selection. Significant correlations were observed between Fst, iHS, and shared ROH regions in bins of 500 kb compared with the whole genome average (p < 0.001) (Additional file 15: Figure S10C and Additional file 16: Figure S11D). We also observed that instead of randomly distributed over the genomes (Additional file 17: Figure S12B), there were several obvious ROH-dense peaks distributed shared between individuals across genomes (Additional file 17: Figure S12A). Therefore, the distribution of ROH is not only result of pure demography, but likely the result of selection.

### Distribution of functional variants in ROH regions

#### Number of deleterious homozygous genotypes in ROH

Additional file 11: Table S2 and Additional file 12: Table S3 show the counts for reference homozygotes (0/0), heterozygotes (0/1), and non-reference homozygotes (1/1) at deleterious and non-deleterious sites, respectively, that were contained within ROH and non-ROH regions (all *g*_*i*,*j*_^*d*,*k*^ and *g*_*i*,*j*_^*n*,*k*^). The number of deleterious non-reference homozygotes was consistent with the ROH coverage in all four breeds. Old-RED and JER showed increased ROH coverage, with a higher number of non-reference deleterious homozygotes in the genome. Non-reference non-deleterious homozygotes also exhibited the same trends as deleterious homozygotes.

**Fig. 3:**
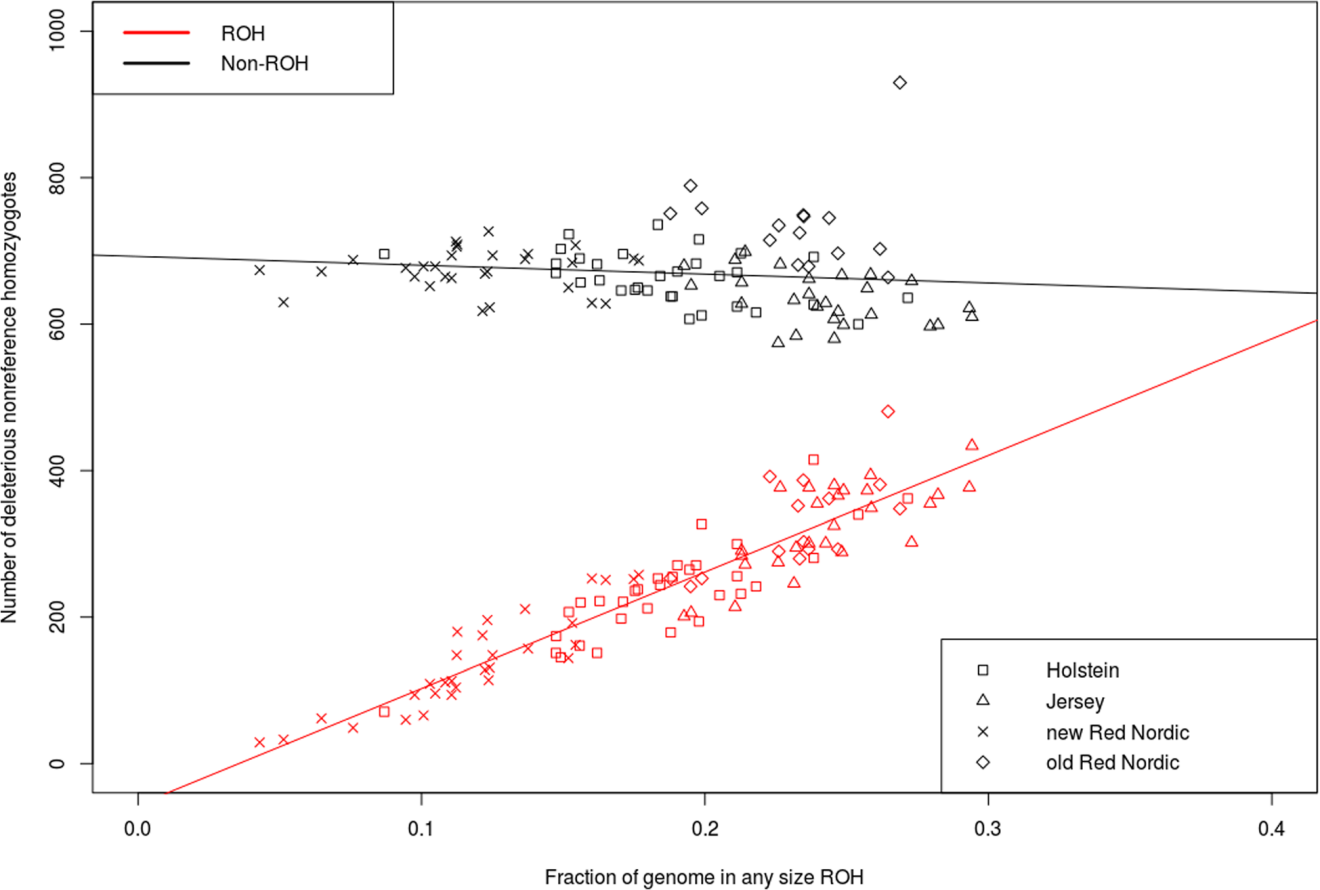
Deleterious non-reference homozygotes versus the genome ROH coverage in each individual. Red points represent the number of deleterious homozygotes falling within ROH regions and black points represent the number of deleterious homozygotes falling outside ROH regions

Figure 3 shows the total number of deleterious non-reference homozygotes (1/1) as a function of the total proportion of the genome covered by ROH (*G*_*i,R*_) for all sequenced individuals. As ROH coverage increased (high *G*_*i,R*_ values), a greater number of homozygotes were observed within ROH, which was consistent with findings from the human genome [24]. There was a very strong positive correlation between the number of deleterious homozygotes and the genomic ROH proportion (Pearson r = 0.93, slope = 1568.76, intercept = −57.63). Similarly, the number of homozygotes outside of ROH decreased with the genomic ROH proportion due to smaller non-ROH regions as ROH coverage increased. As expected, there was a weak negative correlation between deleterious homozygotes outside ROH and the genomic ROH proportions (Pearson r = −0.12, slope = −98.67, intercept = 693.63). Compared with data from the human genome, the decreased slope for non-ROH regions was much shallower than the increased slope for ROH regions [24]. However, this indicates that the increased deleterious homozygotes in ROH regions exceed deleterious homozygote declines in non-ROH regions in cattle. Similar to the human genome [24], the fitted lines also predict that, on average, individual non-inbred cattle (*G*_*i*,*R*_ ≈ 0) carry approximately 694 deleterious homozygous variants. An increased in ROH coverage by 10 % will increase the expected deleterious homozygote numbers in ROH regions by 157 and decrease the expected number of deleterious homozygotes in non-ROH regions by 10, yielding an expected net increase of 147 deleterious homozygotes.

#### Deleterious and non-deleterious homozygotes in ROH of any size

**Fig. 4:**
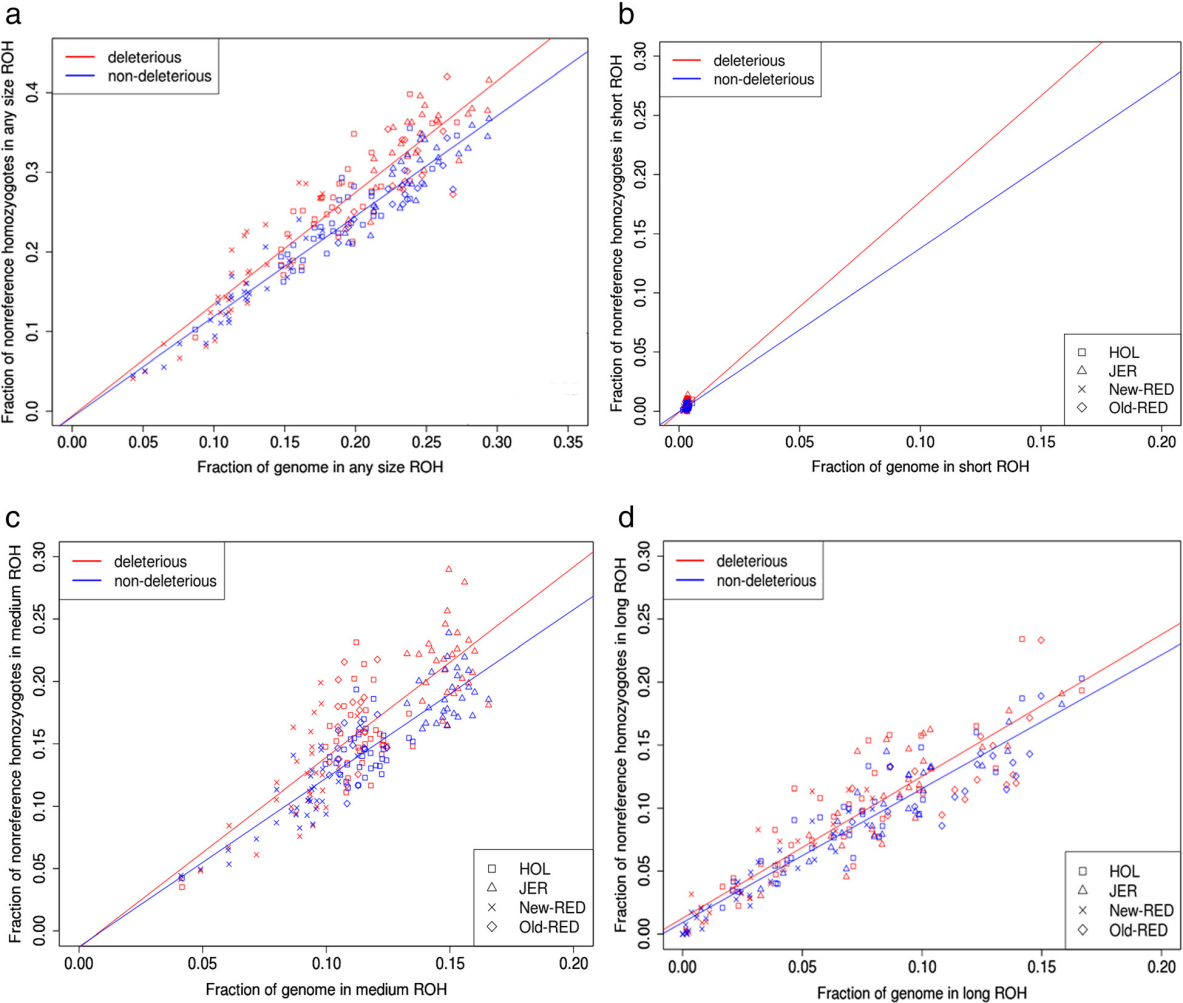
The proportion of all genome-wide non-reference homozygotes falling in ROH regions versus the genome ROH coverage for each individual. **a** Any ROH region; **b** Short; **c** Medium; and **d** Long ROH regions. Red points represent deleterious homozygotes, and blue points represent non-deleterious homozygotes

Figure 4a shows the proportion of deleterious non-reference homozygotes inside and outside ROH regions (*f*_*i*,*R*_^*d*^ and *f*_*i*,*R*_^*n*^) versus total genomic ROH coverage (*G*_*i,R*_). The proportions of non-deleterious and deleterious homozygous genotypes within ROH were strongly positively correlated with total genomic ROH coverage (Pearson r = 0.96 for non-deleterious and r = 0.99 for deleterious). These high correlations were expected, because as larger proportions of homozygous genotypes occur, ROH coverage in the genome increases, and therefore ROH comprise an increasingly greater proportion of the genome [24]. The *f*_*i*,*R*_^*d*^ proportion in genome-wide deleterious homozygotes within ROH consistently exceeded the *f*_*i*,*R*_^*n*^ proportion of genome-wide non-deleterious homozygotes within ROH and the increasing slopes differed between deleterious and non-deleterious variants.

After fitting the two linear regression models, we found *β*_3_ was significant (*P* < 0.05) indicating that the interaction between the two regression slopes (deleterious and non-deleterious) was significant. However, *β*_2_ was not significant (*P* = 0.9174) between the two regression intercepts. This is consistent with previous findings in humans [24], where deleterious homozygotes showed increased frequency in ROH relative to non-deleterious homozygotes and regression slopes were significantly different between deleterious and non-deleterious homozygotes.

#### Deleterious and non-deleterious homozygotes by ROH size

Figure 4b shows *f*_*i*,*S*_^*d*^ and *f*_*i*,*S*_^*n*^ versus total genomic coverage for small ROH (*G*_*i,S*_) (Additional file 6: Figure S6). The proportion of non-deleterious homozygous genotypes in small ROH, and the proportion of deleterious homozygous genotypes in small ROH were positively correlated with genomic coverage (non-deleterious Pearson r = 0.32, deleterious Pearson r = 0.44). Figure 4c and d showed *f*_*i*,*M*_^*d*^ and *f*_*i*,*M*_^*n*^ versus total genomic coverage for medium (*G*_*i,M*_) and large (*G*_*i,L*_) ROH. The regressions for homozygote numbers in medium (non-deleterious r = 0.88, and deleterious r = 0.80) and large (non-deleterious r = 0.94 and deleterious r = 0.90) ROH had smaller *P*-values than in small ROH.

**Fig. 5:**
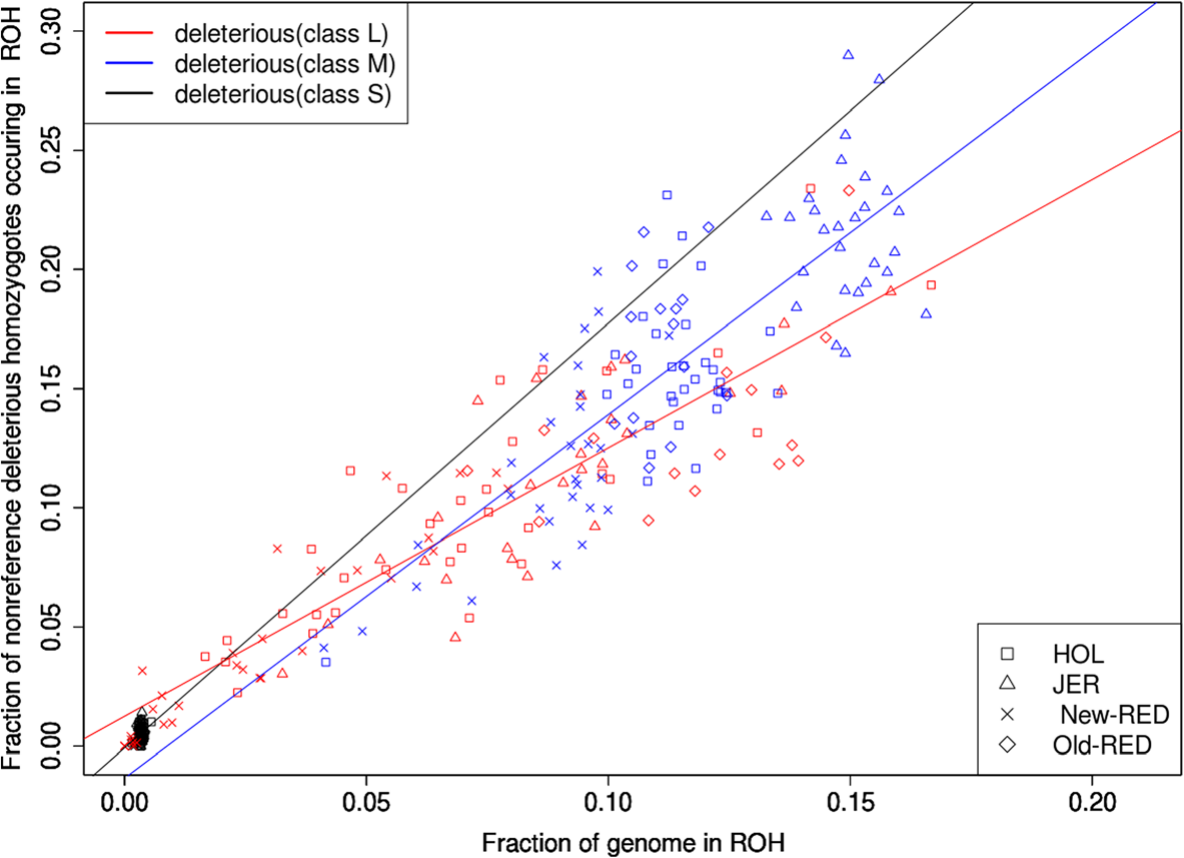
The genome-wide proportion of all non-reference homozygotes falling in different ROH sizes versus genome ROH coverage for each individual. Red, orange, and black points represent deleterious homozygotes in large, medium, and small ROH regions, respectively

These results show that deleterious homozygotes occur more frequently in ROH than non-deleterious homozygotes. Additionally, when the proportion of deleterious homozygotes within large ROH (*f*_*i*,*L*_^*d*^) is compared to the proportion within small ROH (*f*_*i*,*S*_^*d*^), there was a substantially higher proportion of genome-wide deleterious homozygotes in small and medium vs. large ROH especially in individuals with moderate to high ROH coverage proportions (Fig. 5). Given that ROH coverage (Fig. 1) for all individuals across the four breeds differed (as previously mentioned). Therefore, statistical tests for each size group were robust across the breeds and there were different ROH coverage groups across all individuals (Additional file 10: Table S1). Similar trends were observed for each ROH size group, and significantly different degrees of enrichment were observed within each size group.

The intercepts and slopes of deleterious homozygotes and non-deleterious homozygotes were significantly different for large and medium ROH (*β*_2_ = 0.02590, *P* < 0.05; *β*_3_ = −0.39931, *P* < 0.001), but slopes and intercepts were not significantly different between small and medium ROH (*β*_2_ = −0.0126778, *P* = 0.433; *β*_3_ = −0.2562209, *P* = 0.948). These results indicate inbreeding that generates short and medium ROH increases the proportions of deleterious and non-deleterious homozygotes in ROH regions compared to long ROH.

If deleterious, non-deleterious, and synonymous homozygotes are considered as three separate classes, patterns similar to those observed in the deleterious and non-deleterious homozygotes analysis emerge. Deleterious homozygotes were at highest proportions in short and medium length ROH. There were smaller proportions of synonymous and non-deleterious homozygotes as genomic ROH coverage of small and medium ROH increases (Additional file 7: Figure S7 and Additional file 8: Figure S8). In large ROH, the synonymous homozygote proportion over all homozygotes was higher than deleterious and non-deleterious homozygote proportions.

#### Nonsense variants and ROH

**Fig. 6:**
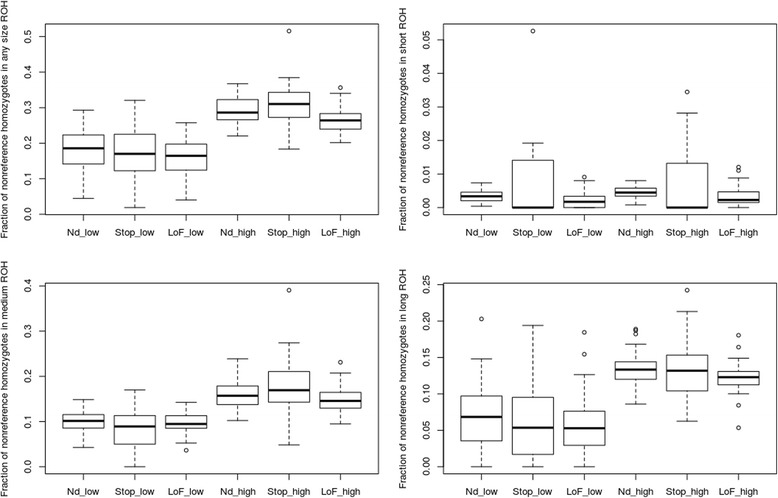
The proportion of all genome-wide non-reference homozygotes falling in ROH regions for non-deleterious variants, nonsense variants, and loss of function nonsense variants versus the genome ROH coverage for individuals in the “low ROH” and “high ROH” groups. A: Any ROH region; B: Short; C: Medium; and D: Long ROH regions

Additional file 13: Table S4 and Additional file 14: Table S5 report nonsense and loss of function nonsense sites, respectively, with counts for reference homozygotes (0/0), heterozygotes (0/1), and non-reference homozygotes (1/1), which fell into ROH and non-ROH regions. Figure 6a shows nonsense mutation distribution across all ROH. For low-ROH individuals, the mean proportion of non-deleterious homozygote variants falling in ROH marginally exceeded the nonsense or loss of function variants. For high-ROH individuals, however, the proportion of non-deleterious homozygotes within ROH was lower than for nonsense homozygotes. When ROH are segregated by size (Fig. 6b–d), including individuals with high genomic ROH coverage, the proportion of nonsense homozygotes in medium ROH was greater than that of non-deleterious homozygotes, while the proportion of nonsense homozygotes was slightly lower than non-deleterious homozygotes for large ROH. This is consistent with the finding that high-ROH individuals exhibited an increased proportion of damaging homozygotes (nonsense mutations) in ROH of any size (Fig. 6a), primarily driven by medium ROH (Fig. 6d).

## Discussion

### Cattle genomic ROH patterns

The 104 individuals re-sequenced in the present study are key ancestors from the four Danish cattle breeds. The New-RED is a composite breed with its primary origins in the Old Danish Red, and includes contributions from other red breeds, including Swedish Red Finnish Ayrshire, and Brown Swiss cattle [37]. Our results showed ROH size ranged from tens of kb to several Mb and varied among individuals from different cattle breeds. Overall, medium sized ROH were most common. The average proportion of the genome represented by ROH was 19.5 % across all sequenced individuals. However, pedigree records and lower density SNP chips underestimated the inbreeding coefficient for these 104 bulls compared with that generated using ROH from genome sequencing. A previous study in Danish cattle also reported a less than 5 % inbreeding coefficient using pedigree information [6]. Among breeds, JER and Old-RED were relatively more inbred compared with HOL based on high ROH genome coverage. Meanwhile, a high number and proportion of long ROH were detected in Old-RED, likely indicating a small population with close mating for some period of time. However, the smallest proportion of ROH was detected in New-RED, presumably due to its outbred origins. These ROH patterns are consistent with the known population history for these four breeds [37]. Pemberton *et al.* allocated humans from different demographics to their corresponding place of origin by inferring population history from ROH analysis [28]. In our study, we also determined that individuals with similar ROH distributions clustered together due to similar patterns of variation (Additional file 5: Figure S5). In contrast, PCA generated different cluster types in the same sets of individuals based on population structure (Additional file 4: Figure S4).

The fact that nucleotide diversity outside ROH was higher in Old-RED than HOL, JER, and New-RED indicates that the historic genetic diversity of Old-RED might be relatively higher than the other breeds examined due to larger breeding populations for each of several past generations. Furthermore, evidence suggested Old-RED was the source population of New-RED. Although the newly derived RED populations exhibited the highest nucleotide diversity across the entire genome, the ancient haplotypes were more diverse in the ancestral Old-RED population. Reduced nucleotide diversity outside ROH in New-RED may also be explained by gene flow from HOL to New-RED. In addition, nucleotide diversity levels outside ROH presumably reflected the different origin of Old-RED from HOL and JER.

Bovine major histocompatibility complex (MHC) regions have long been known in the cattle genome [38, 39]. Our analysis also detected MHC regions, which have a high degree of nucleotide diversity, on chromosome 23 (Additional file 1: Figure S1). Pemberton *et al.* reported a correlation between linkage disequilibrium levels and ROH distribution in the human genome [28]. MHC regions contain recombination hotspots [38, 40] and, therefore, high recombination rates to maintain relatively high levels of genetic diversity, but are subject to over dominant selection [38]. ROH are rarely present in MHC regions of the cattle genome, preventing random distribution of ROH (Additional file 1: Figure S1).

Purfield *et al.* [19] showed ROH patterns in cattle populations using SNP chip data. Our analysis is the first to use next-generation sequencing data to infer ROH in cattle and indicate that long ROH can only be detected with 50 k or HD SNP chip data (minimum ROH size was 1 Mb), while short ROH are not detectable due to the required density of SNP chip data (Additional file 3: Figure S3). Short ROH regions shared between individuals in our data (Additional file 15: Figure S10b and Additional file 16: Fgure S11c) confirm that short ROH were selected and derived from ancient haplotypes that became fixed in populations (Additional file 9: Figure S9), while long ROH are the result of more recent inbreeding events. Consequently, SNP chip data misses information more relevant to historic inbreeding practices rather than recent inbreeding events. The significant correlation between the sharing of ROH regions and regions putatively under selection (from Fst analysis and iHS testing) (Additional file 15: Figure S10C and Additional file 16: Figure S11D) suggests that some of these short shared ROH are the result of a combination of inbreeding and selection. Instead of ROH regions randomly distributed over the genome, there were dense-ROH peak regions in the actual sharing of ROH regions among individuals (Additional file 17: Figure S12), which supports the hypothesis that the observed ROH patterns are not solely a result of demography (Additional file 17: Figure S12), as random ROH distributions would only be expected as a result of inbreeding.

### Distribution of functional variants in the cattle genome

Years of intensive artificial selection in cattle breeding have resulted in reduced genetic diversity in cattle populations as demonstrated by the high proportion of ROH (11.8 – 24.2 %, average 19.5 %) found in this study. However, the results regarding ROH patterns suggest that the distribution and enrichment of putative functional variants in different ROH lengths is more interesting. Szpiech *et al.* suggested that damaging variants are more enriched in human ROH, particularly longer ROH [24]. The observed speed of deleterious homozygote accumulation in ROH far exceeds the accumulated decrease in deleterious homozygotes in non-ROH regions (Fig. 3). This is expected, since identity by decent causes homozygosity to increase in ROH regions compared to non-ROH regions. Deleterious variants were expected at a low frequency; therefore, rare occurrences of deleterious alleles were expected in the homozygous state. However, when a stretch of homologous DNA fragments are identical by descent, the probability of deleterious alleles increases (at a rate of p rather than p^2^, where p is allelic frequency). We also observed a higher allele frequency of deleterious variants in ROH regions compared to non-deleterious variants (Fig. 4). This was also expected as increased deleterious variants occur when allele frequencies are extreme, as has been observed in humans [24]. In cattle, variants with ‘deleterious’ effects on protein structure may be artificially selected more frequently due to economic benefits. One example of this is a myostatin gene mutation in Belgian Blue cattle resulting in a “double muscling” phenotype. Meat from these cattle has a reduced fat content, as the mutation converts feed into increased lean muscle [41].

Cattle populations have been under strong artificial selection for many generations. The significant correlation between sharing of ROH regions and regions putatively under selection pressure (from Fst analysis and iHS testing) (Additional file 15: Figure S10 and Additional file 16: Figure S11) confirms that some of the shared short ROH regions have been selected and spread throughout the population. Moreover, by randomization of ROH regions over the genomes, Additional file 17: Figure S12B presented the patterns of ROH were only result of inbreeding. However, we do observe several dense-ROH peak regions shared among individuals in our populations, further supporting our belief that ROH patterns are not only the result of pure demography. Therefore, the distribution pattern and abundance of functional variants in different ROH lengths in cattle likely differs from the human population. Artificial selection purges deleterious alleles from regions that frequently occur in ROH, favoring alleles with strong beneficial effects. Specifically, the interaction between inbreeding and artificial selection for particular variants can have a strong effect on the distribution of functional variants. Moreover, potentially deleterious mutations might hitchhike with selected variants. Long-term artificial selection enriches cattle populations with beneficial alleles in short and medium ROH, along with hitchhiked deleterious variants, while variants in long ROH remain neutral.

The proportion of predicted deleterious homozygotes was greater in ROH regions than non-deleterious homozygotes. However, predicted deleterious homozygotes varied by the length of ROH. The rate of change differed between small, medium, and long ROH. Higher rates were observed in short and medium compared to long ROH. The slopes for deleterious and non-deleterious homozygotes were significantly different in short and medium ROH, and the patterns were similar for predicted nonsense (gain or loss of a stop codon) and loss of function (in-frame and frameshift) variants (Fig. 6). We also examined patterns of deleterious homozygote frequency in ROH using different length thresholds and saw the similar trends as reported using our original length thresholds (1.small ROH: length < 50 kbp; medium ROH: 50 kbp < =length < =2 Mbp; long ROH: length > 2 Mbp 2. small ROH: length < 150 kbp; medium ROH: 150 kbp < =length < =4 Mbp; long ROH: length > 4 Mbp).

Predicted deleterious variants may be detrimental, however, these allele frequencies cannot increase in long ROH, as inbred individuals harboring a large proportion of long ROH with a high frequency of deleterious alleles will have reduced fitness, leading to decreased survival. Alternatively, predicted deleterious variants may be harmful alleles that were carried into the genome with artificially selected beneficial alleles, and were therefore favored by selection over a number of generations and are reflected in short or medium ROH (Additional file 9: Figure S9, Additional file 15: Figure S10 and Additional file 16: Figure S11). Long ROH are evidence of recent shared ancestry, while short ROH typically reflect more ancient relatedness [30]. Long ROH regions have gone through selection for few generations, and will eventually break down into medium and then short ROH. Allelic combinations will likely be recombined within a few generations and disappear due to segregation [15]. In contrast, deleterious short or medium ROH variants, which reportedly hitchhike with beneficial alleles, are thought to persist for an extended periods of time and are shared among individuals via gene flow (Additional file 9: Figure S9, Additional file 15: Figure S10 and Additional file 16: Figure S11) [15]. Some of these shared short ROH regions were observed to be overlapping with regions under selection based on the Fst analysis and iHS testing (Additional file 15: Figure S10 and Additional file 16: Figure S11). One mechanism for this is when a beneficial mutation occurs in a population and then spreads to the entire population, forming a selective sweep. Artificial selection will favor short or medium ROH regions harboring beneficial mutations that will spread and eventually become fixed in the sampled populations (Additional file 9: Figure S9, Additional file 15: Figure S10 and Additional file 16: Figure S11). Therefore, we deduce that some predicted deleterious homozygotes in short or medium ROH were deleterious alleles that hitchhiked with beneficial variants and were selected in the population. Alternatively, shorter ROH may represent the interplay between random inbreeding and artificial selection for particular variants. Therefore, some of these shared short ROH tended to be candidate regions for selection. However, homozygosity for certain short and medium ROH regions were not maintained due to the absence of selection for any specific alleles; therefore, variants with deleterious effects will be purged by artificial selection. It should also be noted that the confounding effect of inbreeding with selection in generating long stretch of haplotype homozygosity may influence the robustness of EHH-based tests.

Lohmueller *et al.* [29] suggested human populations with decreased genetic diversity supported an excess of recessive deleterious variants, resulting from founder effects in ancient populations during speciation [4], with inflation in the frequency of these rare variants in contemporary populations. We observed a higher proportion of deleterious than non-deleterious homozygotes in ROH (Fig. 4). Therefore, another possible explanation for these results is a history of population inbreeding and founder events (Additional file 9: Figure S9), with preservation of deleterious variants from ancient populations in contemporary cattle populations represented by our samples. However, artificial selection has been implemented in cattle populations for many years, and regions under selection pressure tend to overlap with short, shared ROH regions (Additional file 15: Figure S10 and Additional file 16: Figure S11). This suggests that these preserved ancient alleles may have carried deleterious alleles via hitchhiking.

Predicting how variation affects gene function has varying degrees of reliability. SIFT scores [36] were used to estimate potential fitness consequences for non-reference alleles in our analysis. Certainty regarding predicted functional effects is based on changes in the primary amino acids and impacts on protein function and biological processes. However, here we examined functional variant distribution in ROH regions instead of exploring the effects of each deleterious variant on fitness. A general pattern was obtained by combining all deleterious or non-deleterious variants into one specific class to explore variant distribution in ROH regions. Furthermore, our observations were confirmed by nonsense and loss of function variant classification. Similar patterns were observed when grouping variants into nonsense and loss of function variants. It should be noted we only emphasized non-reference homozygotes with substantial effects on the organism and did not determine the impacts of reference homozygotes. There is the potential for deleterious or selected reference homozygotes, and, therefore, these alleles should also be examined. However, reference alleles are not annotated with a SIFT score, preventing their examination in this study.

## Conclusion

We characterized ROH using genome sequence data in four cattle breeds. The genome-wide proportion and distribution patterns of ROH differed among HOL, JER, New-RED, and Old-RED cattle breeds. We observed a significant correlation between the shared short ROH regions and regions putatively under selection. We also showed the distribution of functional variants in different ROH regions and an increased frequency in predicted deleterious homozygotes in short and medium, but not long, ROH, which differs from the human genome. However, the observed pattern and distribution of functional variants is consistent with the population history of the cattle studied, and we suspect that the observed distribution of functional variants is a result of combination of inbreeding and long-term artificial selection in cattle populations. This is supported by the significant correlation between shared short ROH regions and regions putatively under selection. Our findings contribute to the understanding of the effects of inbreeding and probably selection on shaping the distribution of functional variants in the cattle genome.

## Methods

As previously obtained cattle genomic sequences were used exclusively in this project and no live animal experiments were performed, no animal use and care committee approval was required.

### SNP genotyping, sequencing, variant calling, and quality control

A total of 104 bulls, (*i.e.* 32 HOL, 27 JER, 15 old-RED, and 30 new-RED) with high genetic contributions to the current Danish dairy cattle populations, including Holstein (HOL), Jersey (JER), old Red Danish Dairy cattle (old-RED), and New Danish Red Dairy cattle (new-RED) were selected for sequencing. In addition, 81 and 85 individuals among those sequenced were respectively genotyped using High Density SNP assays (Infinium BovineHD BeadChip), and the 50 k assay Infinium BovineSNP50 v.1 BeadChip (Illumina, San Diego CA). SNP genotyping was performed as described by Höglund *et al.* [42].

All selected individuals’ genomes were sequenced to ~10× depth using Illumina paired-end sequencing. Sequencing was undertaken at the Beijing Genomics (Shenzhen, China), Aros Applied Biotechnology A/S (Aarhus, Denmark), and at Aarhus University (Foulum, Denmark). Reads were aligned to the cattle genome assembly UMD3.1 [43] using *bwa* [44]. Aligned sequences were converted to raw BAM files using *samtools* [45]. Duplicate reads were removed using the *samtools rmdup* option [45]. The Genome Analysis Toolkit [46] was used for local realignment around insertion/deletion (indels) regions, and recalibration following the Human 1000 Genome guidelines incorporating information from *dbSNP* [47]. Finally, variants were called using the *Genome Analysis Toolkit* [46], which simultaneously calls short indels and SNPs by incorporating information from *dbSNP* [48]. Indels were excluded in further analyses, and variants with *phred* scores exceeding 100 were included in nucleotide diversity calculations and ROH computations. Nucleotide diversity was calculated for bins of 10 kbp over the entire genome in all 104 sequenced individuals following the procedures of Bosse *et al.* and Nei and Li [15, 49]. SNP counts per 10 kbp bin were corrected for the number of bases within a 10 kbp bin, which is proportional to 10,000 covered bases. A correction factor must be applied, significant portions (0.5 – 2x) were not covered. The correction factor equaled DP/bin size, where DP is the coverage in bp/bin.

### Principal component analysis

Genotypes were extracted from the sequence data sets (32 HOL, 27 JER, 15 old-RED, and 30 new-RED bulls) following variant calling using a *perl* script. Bi-allelic variant calls with *phred* scores exceeding 100 and higher than average read depths were used in genotype construction. *Genome-wide Complex Trait Analysis* (GCTA) [50] was employed to construct a genetic relationship matrix for chromosome 1 using all sequenced individuals. In addition, the population structure was examined for four breeds using a principal component method [51] using GCTA [50].

### Runs of homozygosity

The method developed by Bosse *et al.* [15] was applied to identify ROH on all autosomes of the 104 sequenced individuals. The threshold to declare a ROH was set to a SNP count maximum of 0.25x the genome coverage following Bosse *et al.* [15]. ROH were also detected in 50 k and HD chip genotyped animals using the Runs of Homozygosity tool in *PLINK* (v. 1.07) [52], with parameters similarly adapted to sequence data. Extracted ROH based on the technique of Bosse *et al.* [15] were compared with ROH calculated from *PLINK* (v. 1.07) [52] for the same individual and chromosome. ROH extracted from sequence data were further classified into three size categories: short ROH are smaller than 100 kbp, and reflect ancient homozygosity haplotypes; medium ROH exhibit sizes from 0.1 to 3 Mbp and arise from relatedness within populations; and long ROH result from recently related individuals, with ROH sized larger than 3 Mbp [15, 30].

The distribution of functional variants in the cattle genome was detected by computing the proportion of an individual’s genome covered by any sized ROH region (j = ROH genome coverage; subscripts denote the ROH size). All comparison tests among breeds were two-tailed *t*-tests performed with the *t.test* function in R (v.3.1.0). Correlation coefficient significance tests were evaluated using the *cor* and *cor.test* function in R (v.3.1.0). The sharing of ROH between individuals among HOL, JER, Old-RED and New-RED was computed by counting the overlap ROH regions between individuals in the 10 kb bin over the full length of genome. To examine if the ROH distribution is the result of pure demography, we randomized the number and length of ROH regions for each individual over the genome and re-distributed them randomly throughout their genomes. These were then compared with the actual sharing of ROH regions between individuals as previously described.

### Detection of selection signatures

#### Fst analysis

The genetic differentiation between individuals among HOL, JER, Old-RED and New-RED was measured by pairwise Fst analysis following Weir and Cockerham [53]. The pairwise Fst between the defined breeds was computed with Genepop 4.2 in bins of 10 kb over the full length of the genome [53]. Correlation between Fst and sharing of ROH averaged for the same bins of 500 kb was calculated with Pearson correlation in R (v.3.1.1).

#### Extended haplotype homozygosity tests

The extended haplotype homozygosity tests were implemented between the breeds for the sequenced individuals. The genome-wide scan for integrated haplotype score (iHS) within each breed was performed using the R package *rehh* [54, 55], and the four breeds were compared using the *ies2rsb* function in *rehh* [54, 56]. Finally, the significance levels (the corresponding p values, assuming iHS or rSB are normally distributed under the neutral hypothesis) between breeds were averaged for a bin of 500 kb and were correlated with ROH sharing for the same bin of 500 kb by Person’s correlation.

### Variant annotations and classifications by predicted functional impacts

The called variants of each genomic site were annotated using ENSEMBL (v.67) databases with *Variant Effect Predictor* (VEP) [35]. Any sites with multiple transcripts resulting in multiple annotations were annotated only once using the *by-gene* option in *VEP* [35]. VEP determines variant effects (*i.e.* SNPs, insertions, deletions, CNVs, or structural variants) on genes, transcripts, and protein sequence, as well as regulatory regions. It predicts genes and transcripts affected by variants, variant locations (*e.g.* upstream of a transcript, in a coding sequence, in non-coding RNA, in regulatory regions), and any variant consequence on protein sequence (*e.g.* gain or loss of a stop codon, missense, frameshift). *SIFT* scores were used to classify annotations for non-reference alleles. Given a set of mutations, *SIFT* predicts the potential effect a non-reference allele has on encoded proteins, and integrates effects of amino acid change, folded structure (predicted or known), and conservation score. *SIFT* categorizes the non-reference mutations as “deleterious” or “tolerated”. In this analysis, we classified non-reference alleles with a “deleterious” predicted effect as “damaging”, while “non-deleterious” or “tolerated” non-reference alleles were classified as “non-damaging”. It should be noted that non-reference alleles predicted as “deleterious” could just be different from reference alleles (in cattle, the reference genome was constructed from a beef breed rather than a dairy breed), which could exhibit substantial effects on amino acid change. Non-reference homozygotes were compared between non-deleterious (non-damaging), deleterious (damaging), and synonymous groups. Although truly damaging alleles could falsely be classified as non-damaging, the objective of this analysis was to detect the distribution of functional variants in regions of homozygosity at the whole genome level.

### Distribution of functional variants in ROH regions

#### Number of deleterious homozygous genotypes in ROH

We followed the method proposed by Szpiech *et al.* [24] to detect predicted functional variant distribution in ROH regions in these three cattle breeds [57]. We partitioned genotypes in our data into those occurring at deleterious versus non-deleterious sites and those occurring outside or inside ROH regions for the given ROH size. Homozygous non-reference genotypes (1/1) in all sequenced individuals were chosen. Alternate non-reference alleles were classified as deleterious or non-deleterious based on predicted effects as previously described [35]. Congruence with Szpiech *et al.* [24] was maintained for individual i, across all sites, by denoting *g*_*i*_^*n*,*k*^ and *g*_*i*_^*d*,*k*^ the total number of sites with k ∈ {0, 1, 2} alternate alleles at non-deleterious and deleterious sites, respectively. For an individual i, *g*_*i*,*j*_^*n*,*k*^ and *g*_*i*,*j*_^*d*,*k*^ represent the total number of sites with k ∈ {0, 1, 2} alternate alleles falling in ROH class j ∈ {S, M, L, R, N} at non-deleterious and deleterious sites, respectively [24]. Here, S, M, and L indicate the different ROH length classes (S: small; M: medium; L: long), R is the union of all three ROH classes, and N represents sites located outside any ROH region [24]. Therefore,

$$ {g}_{i,R}^{n,k}={g}_{i,S}^{n,k}+{g}_{i,M}^{n,k}+{g}_{i,L}^{n,k} $$

$$ {g}_{i,R}^{d,k}={g}_{i,S}^{d,k}+{g}_{i,M}^{d,k}+{g}_{i,L}^{d,k} $$

$$ {g}_{i,R}^{n,k}={g}_i^{n,k}-{g}_{i,R}^{n,k} $$

$$ {g}_{i,N}^{d,k}={g}_i^{d,k}-{g}_{i,R}^{d,k} $$

#### Deleterious and non-deleterious homozygotes in ROH of any size

Following Szpiech *et al.* [24], we compared the proportion of deleterious non-reference homozygotes inside and outside ROH regions to the corresponding proportion of non-deleterious non-reference homozygotes using the formula:

$$ {f}_{i,R}^n=\frac{g_{i,R}^{n,2}}{g_i^{n,2}} $$

where *f*_*i*,*R*_^*n*^ is the proportion of non-deleterious 1/1 homozygotes in individual i that fall in any size ROH. These proportions of non-deleterious 1/1 homozygotes represent the distribution of non-deleterious homozygotes in ROH regions. Similarly, we computed

$$ {f}_{i,R}^d=\frac{g_{i,R}^{d,2}}{g_i^{d,2}} $$

where *f*_*i*,*R*_^*d*^ is the proportion of deleterious 1/1 homozygotes in individual i that fall in any ROH region [24].

We performed two linear regressions on total genomic ROH coverage for deleterious and non-deleterious genotypes, and tested statistical significance of results following Szpiech *et al.* [24]. In addition, we fit a linear model

$$ {f}_{i,R}={\beta}_0+{\beta}_1{G}_{i,R}+{\beta}_2{D}_i+{\beta}_3{G}_{i,R}{D}_i+\varepsilon $$

where *f*_*i*,*R*_ is a vector of length 104 containing, for all individuals, the proportion of genome-wide deleterious homozygotes in any ROH region (*f*_*i*,*R*_^*d*^) and the proportion of genome-wide non-deleterious homozygotes in any ROH region (*f*_*i*,*R*_^*n*^). *G*_*i,R*_ is the proportion of the genome covered by ROH of any size for individual i, and D_i_ is an indicator variable with a value of 1 if the observed response is of deleterious homozygotes or a value of 0 for non-deleterious homozygotes [24]. A statistically significant *β*_2_ (via a two-tailed *t*-test) indicates a difference in the intercepts of separate regressions for deleterious and non-deleterious homozygotes, and a statistically significant *β*_3_ (two-tailed *t*-test) indicates a difference in the regression slopes [24].

#### Deleterious and non-deleterious homozygotes by ROH size class

We subsequently tested how deleterious and non-deleterious homozygotes showed increased frequency in different size classes of ROH regions. It is interesting to explore which ROH lengths (L, M, or S) exhibited increases in deleterious or non-deleterious homozygotes in the cattle genome. Therefore, we separately evaluated each ROH size class following Szpiech *et al.* [24]. Similarly, for homozygous genotypes falling in ROH of size class j (j∈{S, M, L, R, N}), we calculated

$$ {f}_{i,\mathrm{j}}^d=\frac{g_{i,\mathrm{j}}^{d,2}}{g_i^{d,2}} $$

$$ {f}_{i,\mathrm{j}}^n=\frac{g_{i,\mathrm{j}}^{n,2}}{g_i^{n,2}} $$

for deleterious and non-deleterious 1/1 homozygotes, respectively. We investigated data points for each size class for each individual, using the *f*_*i*,*j*_^*d*^ and *f*_*i*,*j*_^*n*^ values.

We tested the statistical difference in these regressions with a linear model analogous to the equations from Szpiech *et al.* [24]. The regression model applied to distinguish deleterious homozygote distributions in ROH size classes is as follows:

$$ {f}_i^d={\beta}_0+{\beta}_1{G}_i+{\beta}_2{C}_i+{\beta}_3{G}_{i,R}{D}_i+\varepsilon $$

where *f*_*i*_^*d*^ is a vector of length 104 containing, for all individuals, the proportions of genome-wide deleterious homozygotes in large ROH (*f*_*i*,*L*_^*d*^), medium ROH (*f*_*i*,*M*_^*d*^), and small ROH (*f*_*i*,*S*_^*d*^). *G*_*i*_ is the proportion of the genome covered by either large (*G*_*i,L*_), medium (*G*_*i,M*_), or small (*G*_*i,S*_) ROH for individual i, and D_i_ is an indicator variable with a value of 1 if the observed response is deleterious homozygotes in large ROH or a value of 0 if the observed response is deleterious homozygotes in small ROH (when comparing large and small ROH). These comparisons were performed between all possible pairings of ROH sizes.

#### Nonsense variants and ROH

Our study classified homozygotes into two predicted classes: deleterious and non-deleterious. Although the deleterious class exhibited increased variants with deleterious effects, a more informative approach would be to examine a subset of variants with an even higher likelihood of being deleterious, *e.g.* nonsense mutations as suggested by Szpiech *et al.* [24], as these are more likely to interfere with normal protein functioning. We tested two sets of predicted nonsense mutations in relationship to their distribution in different ROH lengths. The first set were predicted as stop gain and stop-loss mutations; the second was a mutation set predicted as frame shift and in-frame mutations, which were classified as loss of function variants. Following Szpiech *et al.* [24], we divided individuals into two groups: “low-ROH” and “high-ROH” individuals to examine nonsense variants in ROH regions. Individuals with less than 20 % genomic ROH coverage were classified as low ROH and those with more than 20 % as high ROH.

### Data availability

Data used in this study are from the 1000 Bull Genome Project (Daetwyler *et al.* 2014 Nature Genet. 46:858-865). Whole genome sequence data of individual bulls of the 1000 Bull Genomes Project are already available at NCBI using SRA no. SRP039339 (http://www.ncbi.nlm.nih.gov/bioproject/PRJNA238491).

## Additional files

Additional file 1: Figure S1. Nucleotide diversity distribution on chromosome 23. The X-axis displays the physical position on the chromosome in bp. And the Y-axis shows the corrected SNP number called in 10 kbp bins. Data are from one individual of each breed (HOL, JER, New-RED, and old-RED). Additional file 2: Figure S2. Heterozygosity distribution as log_2_ (number of SNPs) per 10 kbp from one individual of each cattle breed (New-RED: blue; HOL: green; JER: red; and Old-RED: orange). The plot only shows bins with heterozygosity greater than 0. Additional file 3: Figure S3. ROH detected from sequence data, HD SNP, and 50 k SNP chip data for chromosome 1 from one Holstein. The X-axis displays the physical position on the chromosome in bp. The Y-axis shows the corrected number of SNPs called in 10 kbp bins. The grey shadows indicate ROH location called from sequence data, HD SNP, and 50 k SNP chip data. Additional file 4: Figure S4. Principal component analysis (PCA) depicts the population structure derived from patterns of variability for sequenced individuals from four cattle breeds (HOL: yellow; JER: green; Old-RED: blue; and New-RED: red). Additional file 5: Figure S5. Three-point ROH statistics for all 104 sequenced individuals. The number of ROH in the genome of each individual is plotted on the X-axis, the average ROH size (bp) is depicted on the Y-axis, and nucleotide diversity in a 10 kb window is shown on the Z-axis (HOL: yellow; JER: green; Old-RED: blue; and New-RED: red). Additional file 6: Figure S6. The proportion of all genome-wide non-reference homozygotes falling in short ROH regions versus the genome ROH coverage for each individual. Red points represent deleterious homozygotes, and orange points represent non-deleterious homozygotes. Additional file 7: Figure S7. The genome-wide proportion of all non-reference homozygotes falling in ROH regions versus the genome ROH coverage for each individual. A: Any ROH region; B: Short; C: Medium; and D: Long ROH regions. Red points represent deleterious homozygotes, orange points represent non-deleterious homozygotes, and black points represent synonymous homozygotes. Additional file 8: Figure S8. The proportion of all genome-wide non-reference homozygotes falling in short ROH regions versus the genome short ROH coverage for each individual. Red points represent deleterious homozygotes, orange points represent non-deleterious homozygotes, and black points represent synonymous homozygotes. Additional file 9: Figure S9. ROH distribution on chromosome 6. The X-axis plots ROH on chromosome 6 and the Y-axis represents each of the 104 individuals. Lines indicate ROH segments across the genome. Red represents HOL, blue JER, brown Old-RED, and purple represents New-RED. Additional file 10: Table S1. Summary statistics of all sequenced cattle of the Holstein, Jersey, Danish New-red and Danish old-red breeds. The column “Animal” shows the codes for each individual. The ROH proportion across the genome is shown in column 2. Columns 3, 4, and 5 display the average ROH size (bp), number of ROHs, and total length of ROH (bp) in the genome, respectively. Nucleotide diversity across the whole genome and outside ROH are shown in columns 6 and 7. The last three columns display the proportion of short, medium and long ROH. Additional file 11: Table S2. Genotype counts per cattle individual for sites classified as deleterious. Additional file 12: Table S3. Genotype counts per individual for sites classified as non-deleterious. Additional file 13: Table S4. Genotype counts per individual for sites classified as stop gain and stop-loss mutations (nonsense). Additional file 14: Table S5. Genotype counts per individual for sites classified as frame shift and in-frame mutations (loss of function). Additional file 15: Figure S10. Signatures of selection based on Fst analysis in sequenced populations. A. Fst values (y-axis) are plotted for each 10 kb bin for all 29 chromosomes. B. The sharing of ROH regions in a 10 kb bin among all individuals for all chromosomes; the y-axis is the number of individuals who have the same region of ROH in a 10 kb bin. C. Correlation between averaged Fst values (y-axis) and number of individuals sharing the same ROH regions in a 500 kb bin (x-axis) (Pearson’s product-moment correlation coefficient = 0.20; *p* < 0.001). Additional file 16: Figure S11. Signatures of selection based on iHS testing in sequenced populations. A. The iHS signals (y-axis) between sequenced populations are plotted for all 29 chromosomes. B. Significance of the rSB signals (corresponding p values) for sequenced populations for each chromosome. C. The sharing of ROH regions in a 10 kb bin among all individuals for all chromosomes; the y-axis is the number of individuals who have the same ROH region in a 10 kb bin. D. Correlation between p values of iHS signals (y-axis) and number of individuals sharing the same ROH regions in a 500 kb bin (x-axis) (Pearson’s product-moment correlation coefficient = 0.22; *p* < 0.001). Additional file 17: Figure S12. Permutation of ROH regions compared with sharing of ROH regions among individuals. A. Sharing of ROH regions in a 10 kb bin among all individuals for all chromosomes; the y-axis is the number of individuals who have the same ROH region in a 10 kb bin. B. The randomized ROH regions over genomes in 10 kb bins; the y-axis is the number of individuals who have the same ROH region in a 10 kb bin.

## Abbreviations

ROH: Runs of homozygosity
HOL: Holstein cattle
JER: Jersey cattle
New-RED: New Danish red cattle
Old-RED: Old Danish red cattle
VEP: Variant Effect Predictor
CNV: Copy number variation
MHC: Bovine major histocompatibility complex
IBD: Identity by descent
PCA: Principal component analysis
LOF: Loss of function

## References

[1] Hayes BJ, Bowman PJ, Chamberlain AJ, Goddard ME (2009). Invited review: Genomic selection in dairy cattle: progress and challenges (vol 92, pg 433, 2009). J Dairy Sci.

[2] Brade W, Brade E. Breeding History of German Holstein Cattle. Ber Landwirtsch 2013, 91(2). 10.12767/buel.v91i2.25.g70.

[3] Christensen LG (1989). Cattle-Breeding after 1992. Zuchtungskunde.

[4] Ramachandran S, Deshpande O, Roseman CC, Rosenberg NA, Feldman MW, Cavalli-Sforza LL (2005). Support from the relationship of genetic and geographic distance in human populations for a serial founder effect originating in Africa. Proc Natl Acad Sci U S A.

[5] Wright S (1921). Systems of mating. II. The effects of inbreeding on the genetic composition of a population. Genetics.

[6] Sorensen AC, Sorensen MK, Berg P (2005). Inbreeding in Danish dairy cattle breeds. J Dairy Sci.

[7] Bjelland DW, Weigel KA, Vukasinovic N, Nkrumah JD (2013). Evaluation of inbreeding depression in Holstein cattle using whole-genome SNP markers and alternative measures of genomic inbreeding. J Dairy Sci.

[8] Nomura T, Honda T, Mukai F (2001). Inbreeding and effective population size of Japanese Black cattle. J Anim Sci.

[9] Miglior F, Muir BL, Van Doormaal BJ (2005). Selection indices in Holstein cattle of various countries. J Dairy Sci.

[10] Gonzalez-Recio O, de Maturana EL, Gutierrez JP (2007). Inbreeding depression on female fertility and calving ease in Spanish dairy cattle. J Dairy Sci.

[11] Freyer G, Hernandez-Sanchez J, Cassell BG (2005). A note on inbreeding in dairy cattle breeding. Arch Tierzucht.

[12] Ku CS, Naidoo N, Teo SM, Pawitan Y (2011). Regions of homozygosity and their impact on complex diseases and traits. Hum Genet.

[13] Miglior F, Szkotnicki B, Burnside EB (1992). Analysis of Levels of Inbreeding and Inbreeding Depression in Jersey Cattle. J Dairy Sci.

[14] Weigel K (2006). Controlling inbreeding in modern dairy breeding programs. Adv Dairy Technol.

[15] Bosse M, Megens HJ, Madsen O, Paudel Y, Frantz LA, Schook LB (2012). Regions of homozygosity in the porcine genome: consequence of demography and the recombination landscape. PLoS Genet.

[16] Pusey A, Wolf M (1996). Inbreeding avoidance in animals. Trends Ecol Evol.

[17] Koenig S, Simianer H (2006). Approaches to the management of inbreeding and relationship in the German Holstein dairy cattle population. Livest Sci.

[18] Margolin S, Bartlett JW (1945). The Influence of Inbreeding Upon the Weight and Size of Dairy Cattle. J Anim Sci.

[19] Purfield DC, Berry DP, McParland S, Bradley DG. Runs of homozygosity and population history in cattle. BMC Genet. 2012;13. 10.1186/1471-2156-13-70 PMC3502433 22888858

[20] Lencz T, Lambert C, DeRosse P, Burdick KE, Morgan TV, Kane JM (2007). Runs of homozygosity reveal highly penetrant recessive loci in schizophrenia. Proc Natl Acad Sci U S A.

[21] Nalls MA, Guerreiro RJ, Simon-Sanchez J, Bras JT, Traynor BJ, Gibbs JR (2009). Extended tracts of homozygosity identify novel candidate genes associated with late-onset Alzheimer’s disease. Neurogenetics.

[22] Charlesworth B, Morgan MT, Charlesworth D (1993). The Effect of Deleterious Mutations on Neutral Molecular Variation. Genetics.

[23] Lohmueller KE, Albrechtsen A, Li YR, Kim SY, Korneliussen T, Vinckenbosch N, et al. Natural Selection Affects Multiple Aspects of Genetic Variation at Putatively Neutral Sites across the Human Genome. PLoS Genetics 2011, 7(10). doi:10.1371/journal.pgen.1002326. 10.1371/journal.pgen.1002326 PMC3192825 22022285

[24] Szpiech ZA, Xu JS, Pemberton TJ, Peng WP, Zollner S, Rosenberg NA (2013). Long Runs of Homozygosity Are Enriched for Deleterious Variation. Am J Hum Genet.

[25] Loftus RT, MacHugh DE, Bradley DG, Sharp PM, Cunningham P (1994). Evidence for two independent domestications of cattle. Proc Natl Acad Sci U S A.

[26] Kim ES, Cole JB, Huson H, Wiggans GR, Van Tassell CP, Crooker BA et al. Effect of Artificial Selection on Runs of Homozygosity in US Holstein Cattle. Plos One 2013, 8(11). doi:10.1371/journal.pone.0080813 10.1371/journal.pone.0080813 PMC3858116 24348915

[27] Leocard S (2009). Selective Sweep and the Size of the Hitchhiking Set. Adv Appl Probab.

[28] Pemberton TJ, Absher D, Feldman MW, Myers RM, Rosenberg NA, Li JZ (2012). Genomic patterns of homozygosity in worldwide human populations. Am J Hum Genet.

[29] Lohmueller KE, Indap AR, Schmidt S, Boyko AR, Hernandez RD, Hubisz MJ (2008). Proportionally more deleterious genetic variation in European than in African populations. Nature.

[30] Kirin M, McQuillan R, Franklin CS, Campbell H, McKeigue PM, Wilson JF. Genomic Runs of Homozygosity Record Population History and Consanguinity. Plos One 2010, 5(11). doi:10.1371/journal.pone.0013996 10.1371/journal.pone.0013996 PMC2981575 21085596

[31] Carbone I, Jakobek JL, Ramirez-Prado JH, Horn BW (2007). Recombination, balancing selection and adaptive evolution in the aflatoxin gene cluster of Aspergillus parasiticus. Mol Ecol.

[32] Jansen S, Aigner B, Pausch H, Wysocki M, Eck S, Benet-Pages A, et al. Assessment of the genomic variation in a cattle population by re-sequencing of key animals at low to medium coverage. BMC Genomics. 2013;14. 10.1186/1471-2164-14-446 PMC3716689 23826801

[33] Zhan BJ, Fadista J, Thomsen B, Hedegaard J, Panitz F, Bendixen C. Global assessment of genomic variation in cattle by genome resequencing and high-throughput genotyping. BMC Genomics. 2011;12. 10.1186/1471-2164-12-557 PMC3248099 22082336

[34] Stothard P, Choi JW, Basu U, Sumner-Thomson JM, Meng Y, Liao XP, et al. Whole genome resequencing of Black Angus and Holstein cattle for SNP and CNV discovery. BMC Genomics. 2011;12. 10.1186/1471-2164-12-559 PMC3229636 22085807

[35] McLaren W, Pritchard B, Rios D, Chen YA, Flicek P, Cunningham F (2010). Deriving the consequences of genomic variants with the Ensembl API and SNP Effect Predictor. Bioinformatics.

[36] Velankar S, Dana JM, Jacobsen J, van Ginkel G, Gane PJ, Luo J (2013). SIFTS: Structure Integration with Function, Taxonomy and Sequences resource. Nucleic Acids Res.

[37] Andersen B, Jensen B, Nielsen A, Christensen LG, Liboriussen T. Rød Dansk Malkerace-avlsmæssigt of kulturhistorisk belyst. Danmarks HordbrugsForskning. Denmark. 2003.

[38] Andersson L, Lunden A, Sigurdardottir S, Davies CJ, Rask L (1988). Linkage Relationships in the Bovine Mhc Region - High Recombination Frequency between Class-Ii Subregions. Immunogenetics.

[39] Ellis SA, Ballingall KT (1999). Cattle MHC: evolution in action?. Immunol Rev.

[40] Kauppi L, Sajantila A, Jeffreys AJ (2003). Recombination hotspots rather than population history dominate linkage disequilibrium in the MHC class II region. Hum Mol Genet.

[41] Kambadur R, Sharma M, Smith TPL, Bass JJ (1997). Mutations in myostatin (GDF8) in double-muscled Belgian blue and Piedmontese cattle. Genome Res.

[42] Hoglund JK, Sahana G, Guldbrandtsen B, Lund MS. Validation of associations for female fertility traits in Nordic Holstein, Nordic Red and Jersey dairy cattle. BMC Genetics 2014, 15. doi:10.1186/1471-2156-15-8 10.1186/1471-2156-15-8 PMC3898023 24428918

[43] Zimin AV, Delcher AL, Florea L, Kelley DR, Schatz MC, Puiu D et al. A whole-genome assembly of the domestic cow, Bos taurus. Genome Biol 2009, 10(4). doi:10.1186/gb-2009-10-4-r42 10.1186/gb-2009-10-4-r42 PMC2688933 19393038

[44] Li H, Durbin R (2009). Fast and accurate short read alignment with Burrows-Wheeler transform. Bioinformatics.

[45] Li H, Handsaker B, Wysoker A, Fennell T, Ruan J, Homer N (2009). The Sequence Alignment/Map format and SAMtools. Bioinformatics.

[46] McKenna A, Hanna M, Banks E, Sivachenko A, Cibulskis K, Kernytsky A (2010). The Genome Analysis Toolkit: a MapReduce framework for analyzing next-generation DNA sequencing data. Genome Res.

[47] Sherry ST, Ward MH, Kholodov M, Baker J, Phan L, Smigielski EM (2001). dbSNP: the NCBI database of genetic variation. Nucleic Acids Res.

[48] Sherry ST, Ward MH, Kholodov M, Baker J, Phan L, Smigielski EM (2000). dbSNP: the NCBI database of genetic variation. Nucleic Acids Res.

[49] Nei M, Li WH (1979). Mathematical-Model for Studying Genetic-Variation in Terms of Restriction Endonucleases. Proc Natl Acad Sci U S A.

[50] Yang JA, Lee SH, Goddard ME, Visscher PM (2011). GCTA: A Tool for Genome-wide Complex Trait Analysis. Am J Hum Genet.

[51] Price AL, Patterson NJ, Plenge RM, Weinblatt ME, Shadick NA, Reich D (2006). Principal components analysis corrects for stratification in genome-wide association studies. Nat Genet.

[52] Purcell S, Neale B, Todd-Brown K, Thomas L, Ferreira MAR, Bender D (2007). PLINK: A tool set for whole-genome association and population-based linkage analyses. Am J Hum Genet.

[53] Weir BS, Cockerham CC (1984). Estimating F-Statistics for the Analysis of Population-Structure. Evolution.

[54] Gautier M, Vitalis R (2012). rehh: an R package to detect footprints of selection in genome-wide SNP data from haplotype structure. Bioinformatics.

[55] Sabeti PC, Reich DE, Higgins JM, Levine HZP, Richter DJ, Schaffner SF (2002). Detecting recent positive selection in the human genome from haplotype structure. Nature.

[56] Tang K, Thornton KR, Stoneking M (2007). A new approach for using genome scans to detect recent positive selection in the human genome. PLos Biol.

[57] Zhang Q, Calus M, Guldbrandtsen B, Lund MS, Sahana G. Estimation of inbreeding using pedigree, 50k SNP chip genotypes and full sequence data in three cattle breeds. BMC Genetics, in press. 2015. 10.1186/s12863-015-0227-7 PMC4509611 26195126

