# Supplementary figures and images for: Runs of homozygosity and distribution of functional variants in the cattle genome
Source: BMC Genomics. 2015 Jul 22;16(1):542. doi: 10.1186/s12864-015-1715-x (PMC4508970; doi:10.1186/s12864-015-1715-x)

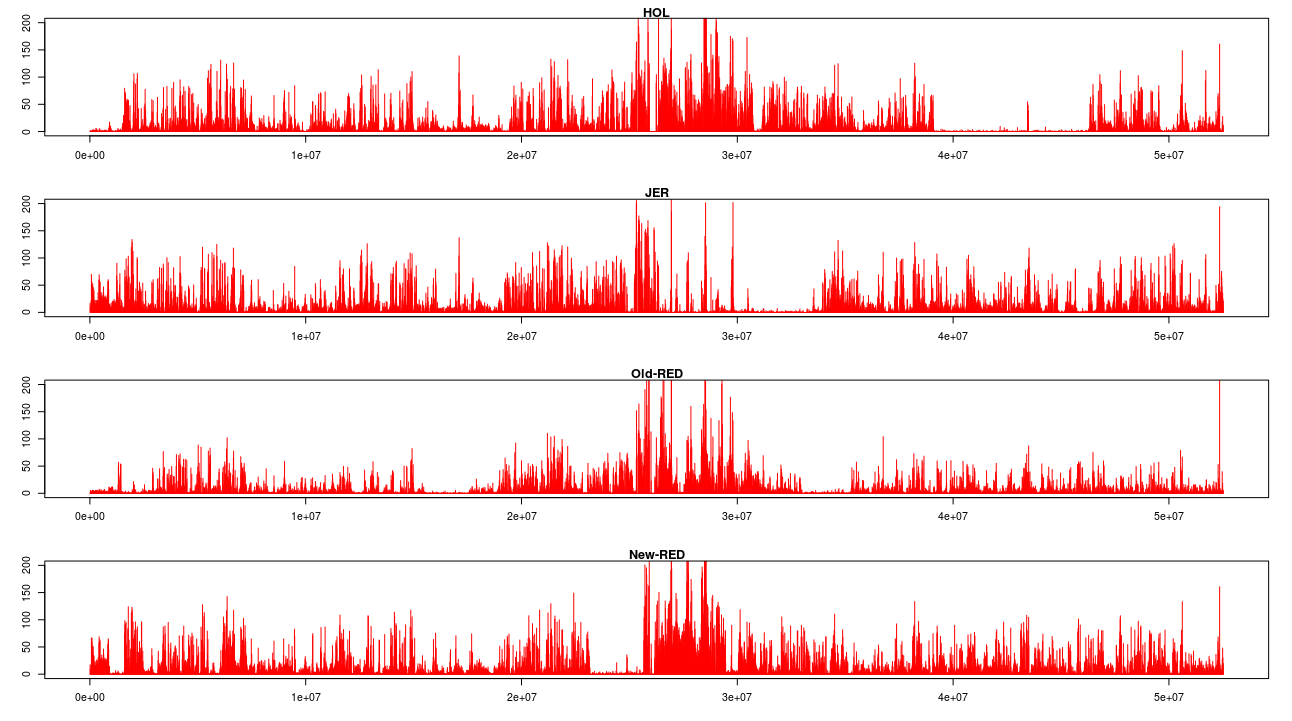

Supplement: Additional file 1: Figure S1. — Nucleotide diversity distribution on chromosome 23. The X-axis displays the physical position on the chromosome in bp. And the Y-axis shows the corrected SNP number called in 10 kbp bins. Data are from one individual of each breed (HOL, JER, New-RED, and old-RED). [file 12864_2015_1715_MOESM1_ESM.tiff]

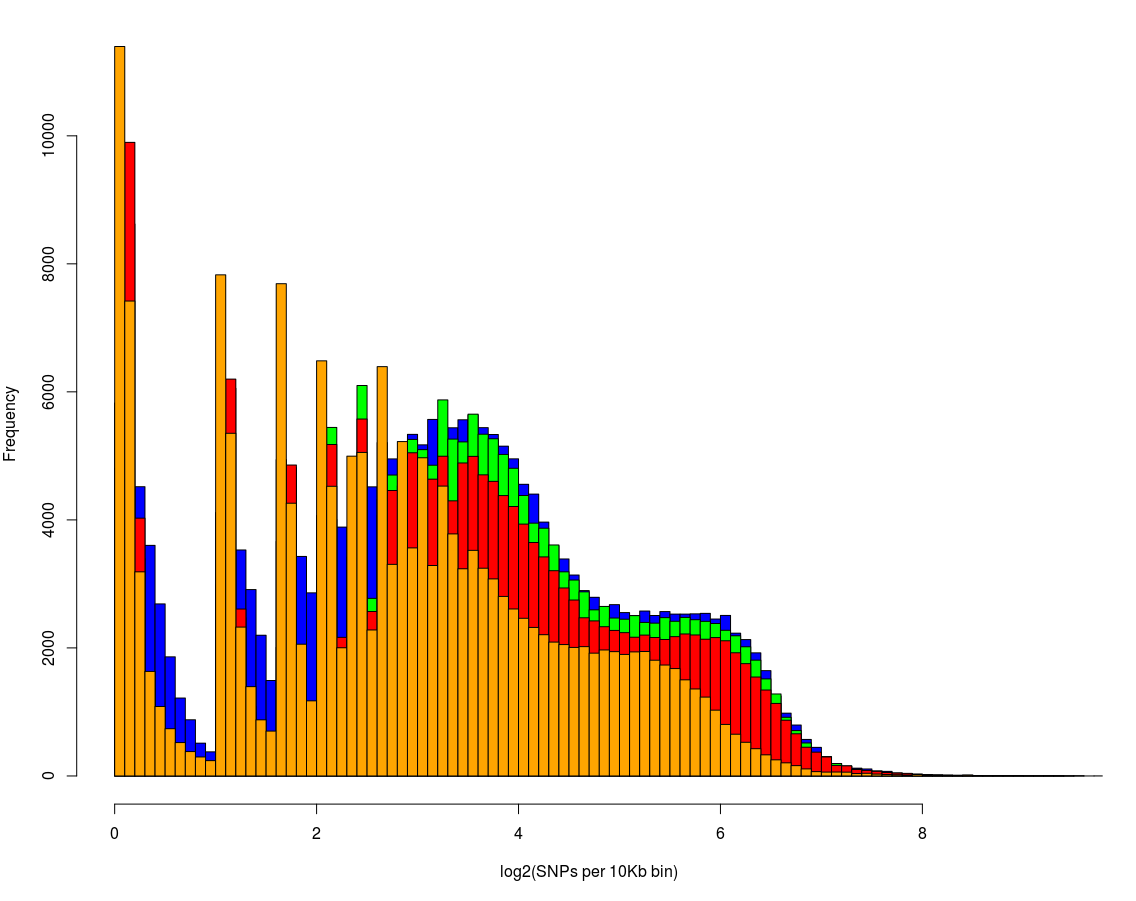

Supplement: Additional file 2: Figure S2. — Heterozygosity distribution as log2 (number of SNPs) per 10 kbp from one individual of each cattle breed (New-RED: blue; HOL: green; JER: red; and Old-RED: orange). The plot only shows bins with heterozygosity greater than 0. [file 12864_2015_1715_MOESM2_ESM.tiff]

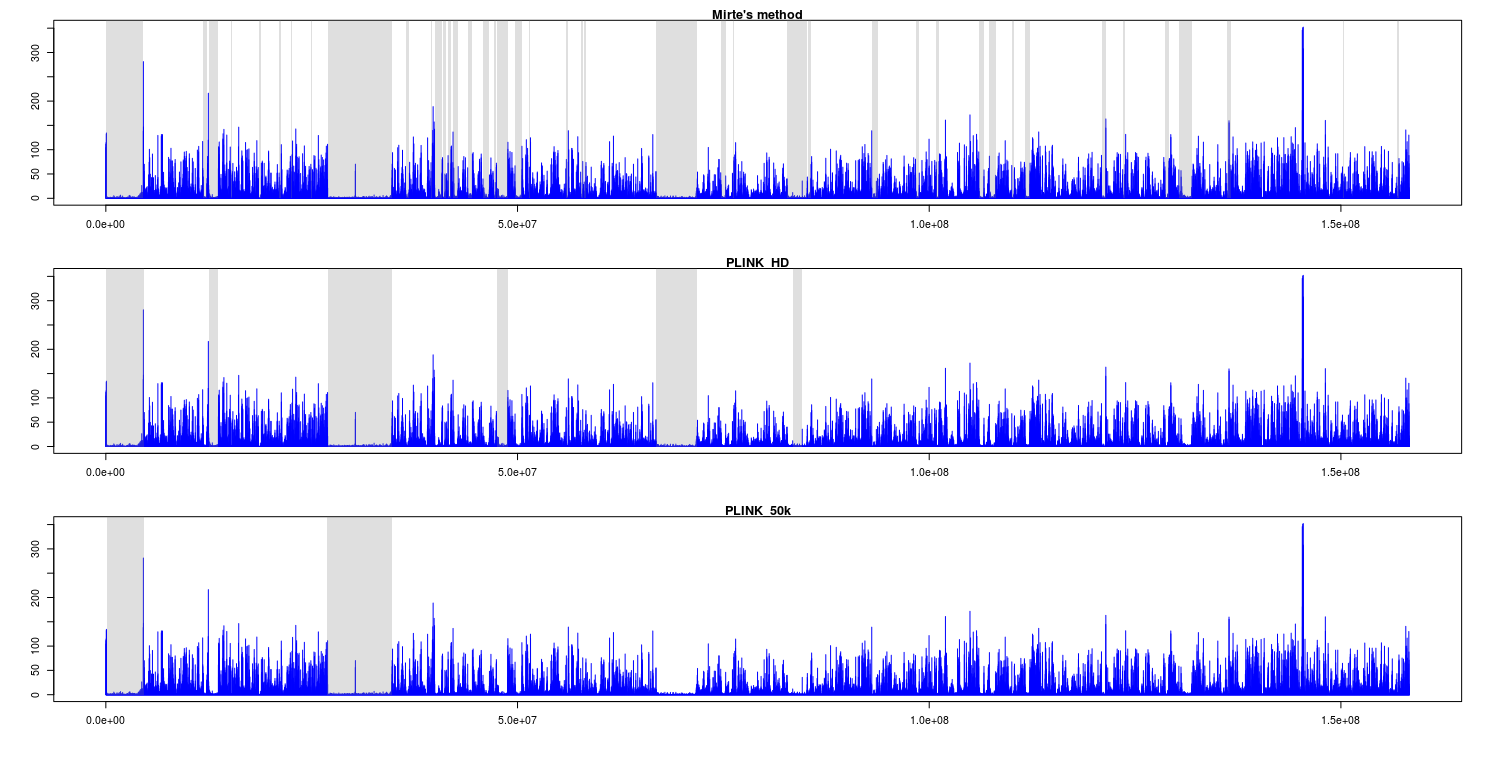

Supplement: Additional file 3: Figure S3. — ROH detected from sequence data, HD SNP, and 50 k SNP chip data for chromosome 1 from one Holstein. The X-axis displays the physical position on the chromosome in bp. The Y-axis shows the corrected number of SNPs called in 10 kbp bins. The grey shadows indicate ROH location called from sequence data, HD SNP, and 50 k SNP chip data. [file 12864_2015_1715_MOESM3_ESM.tiff]

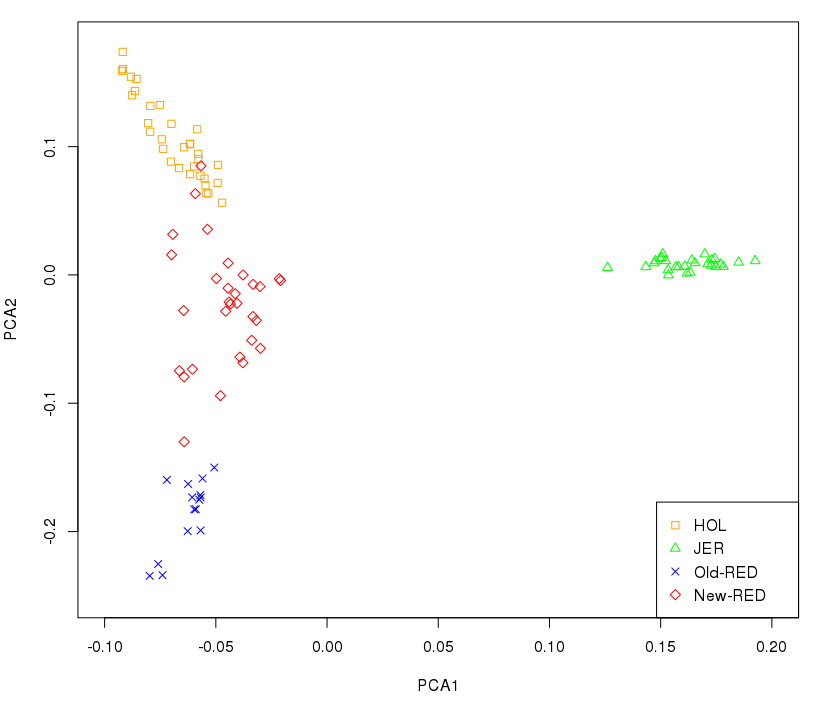

Supplement: Additional file 4: Figure S4. — Principal component analysis (PCA) depicts the population structure derived from patterns of variability for sequenced individuals from four cattle breeds (HOL: yellow; JER: green; Old-RED: blue; and New-RED: red). [file 12864_2015_1715_MOESM4_ESM.tiff]

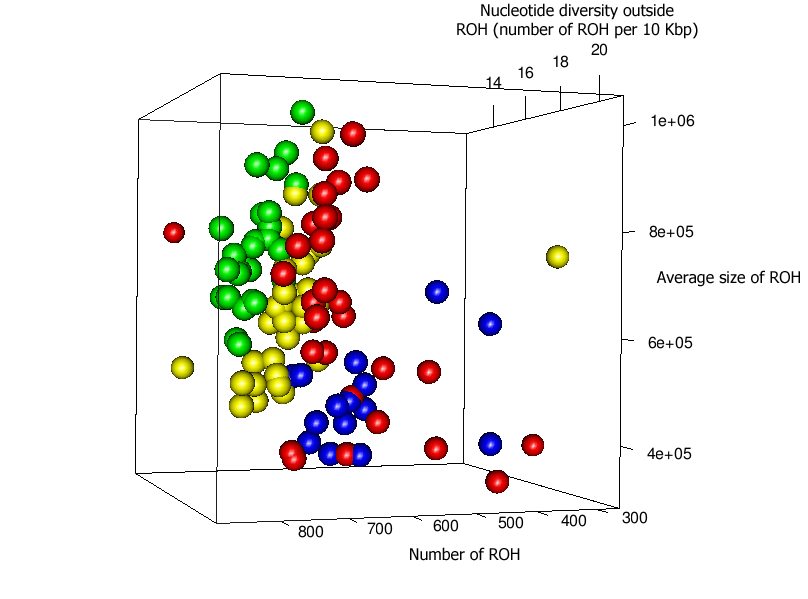

Supplement: Additional file 5: Figure S5. — Three-point ROH statistics for all 104 sequenced individuals. The number of ROH in the genome of each individual is plotted on the X-axis, the average ROH size (bp) is depicted on the Y-axis, and nucleotide diversity in a 10 kb window is shown on the Z-axis (HOL: yellow; JER: green; Old-RED: blue; and New-RED: red). [file 12864_2015_1715_MOESM5_ESM.tiff]

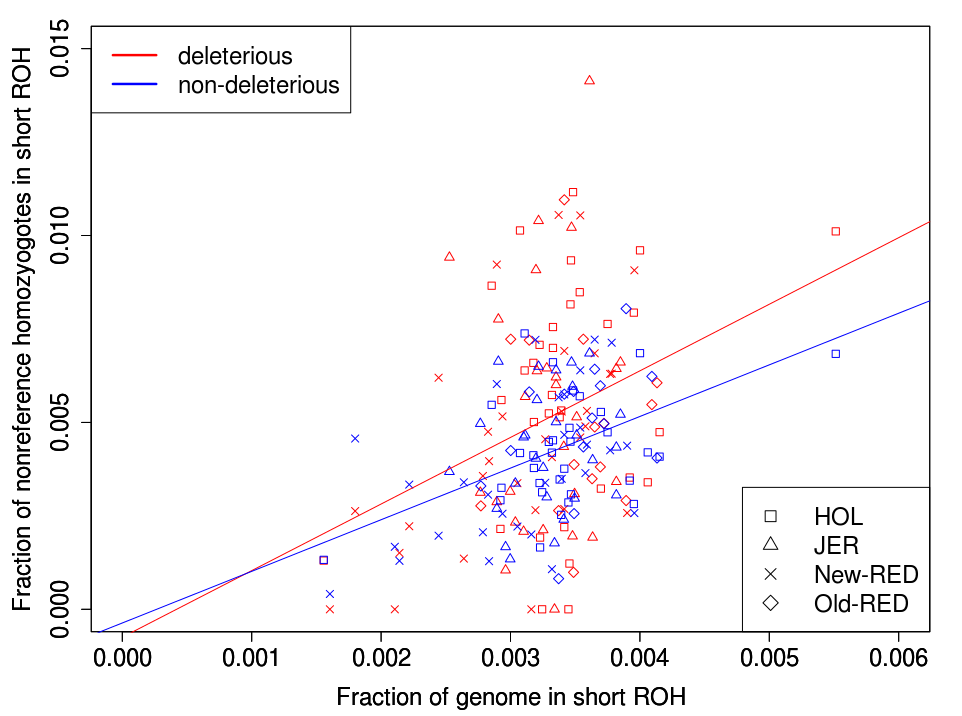

Supplement: Additional file 6: Figure S6. — The proportion of all genome-wide non-reference homozygotes falling in short ROH regions versus the genome ROH coverage for each individual. Red points represent deleterious homozygotes, and orange points represent non-deleterious homozygotes. [file 12864_2015_1715_MOESM6_ESM.tiff]

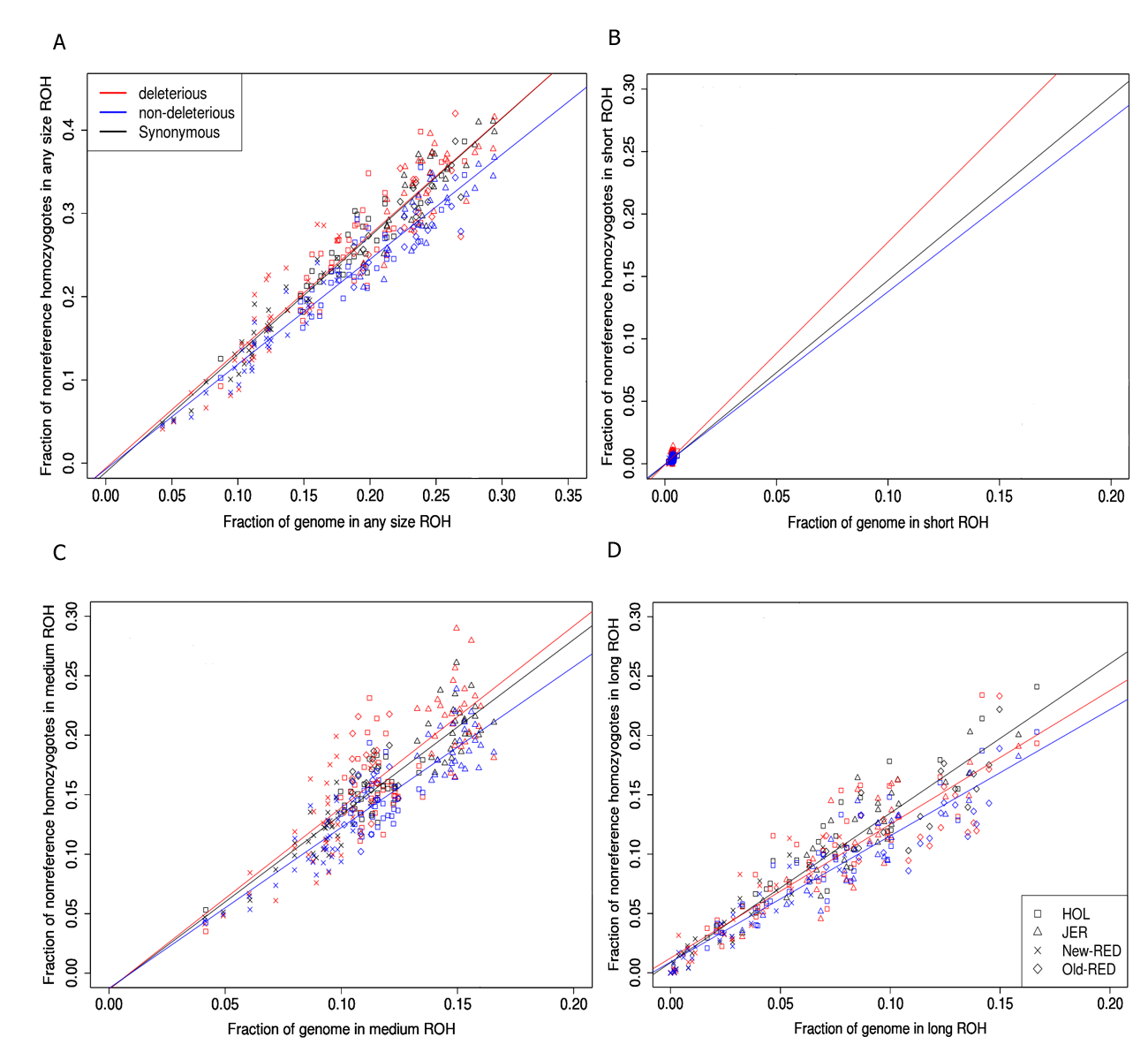

Supplement: Additional file 7: Figure S7. — The genome-wide proportion of all non-reference homozygotes falling in ROH regions versus the genome ROH coverage for each individual. A: Any ROH region; B: Short; C: Medium; and D: Long ROH regions. Red points represent deleterious homozygotes, orange points represent non-deleterious homozygotes, and black points represent synonymous homozygotes. [file 12864_2015_1715_MOESM7_ESM.tiff]

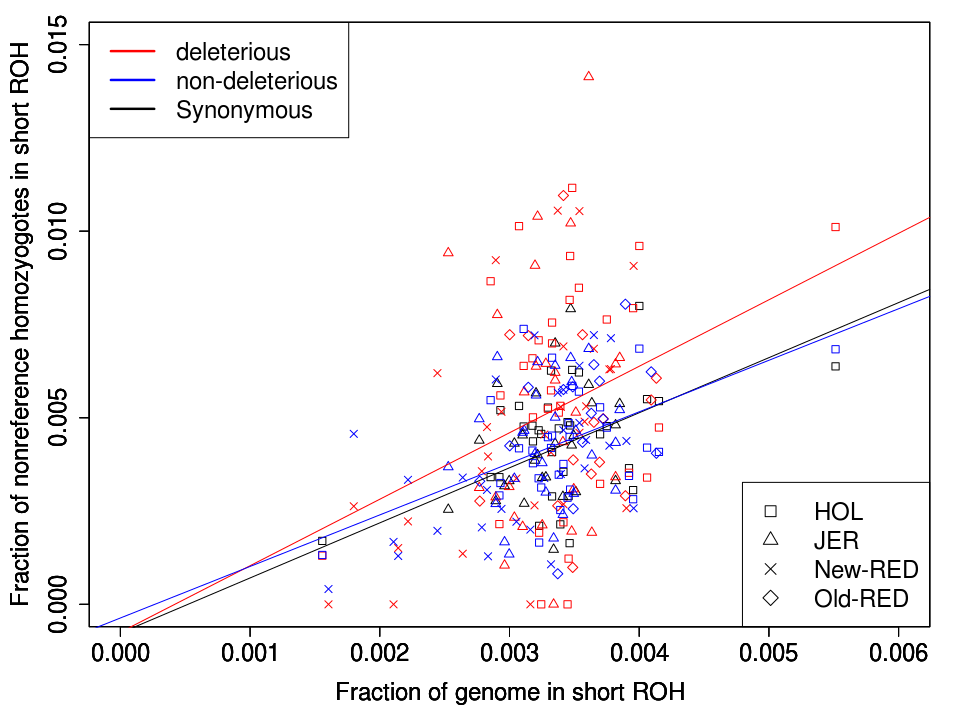

Supplement: Additional file 8: Figure S8. — The proportion of all genome-wide non-reference homozygotes falling in short ROH regions versus the genome short ROH coverage for each individual. Red points represent deleterious homozygotes, orange points represent non-deleterious homozygotes, and black points represent synonymous homozygotes. [file 12864_2015_1715_MOESM8_ESM.tiff]

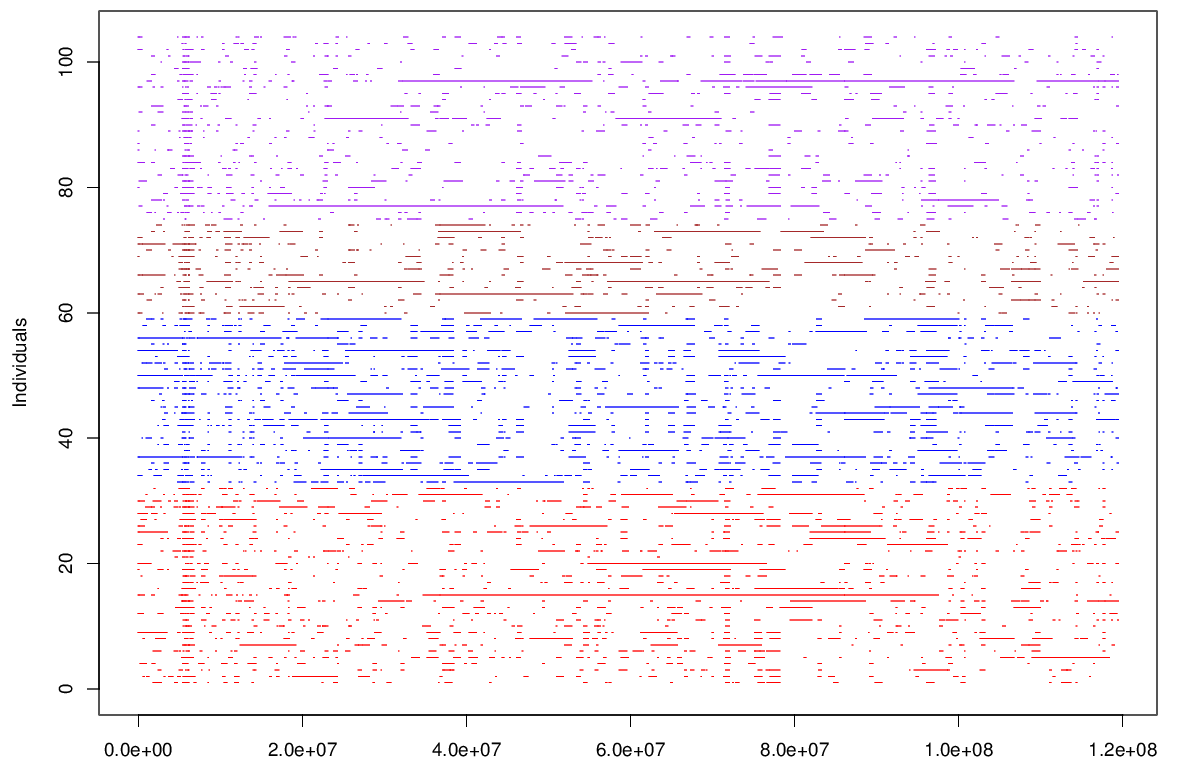

Supplement: Additional file 9: Figure S9. — ROH distribution on chromosome 6. The X-axis plots ROH on chromosome 6 and the Y-axis represents each of the 104 individuals. Lines indicate ROH segments across the genome. Red represents HOL, blue JER, brown Old-RED, and purple represents New-RED. [file 12864_2015_1715_MOESM9_ESM.tiff]

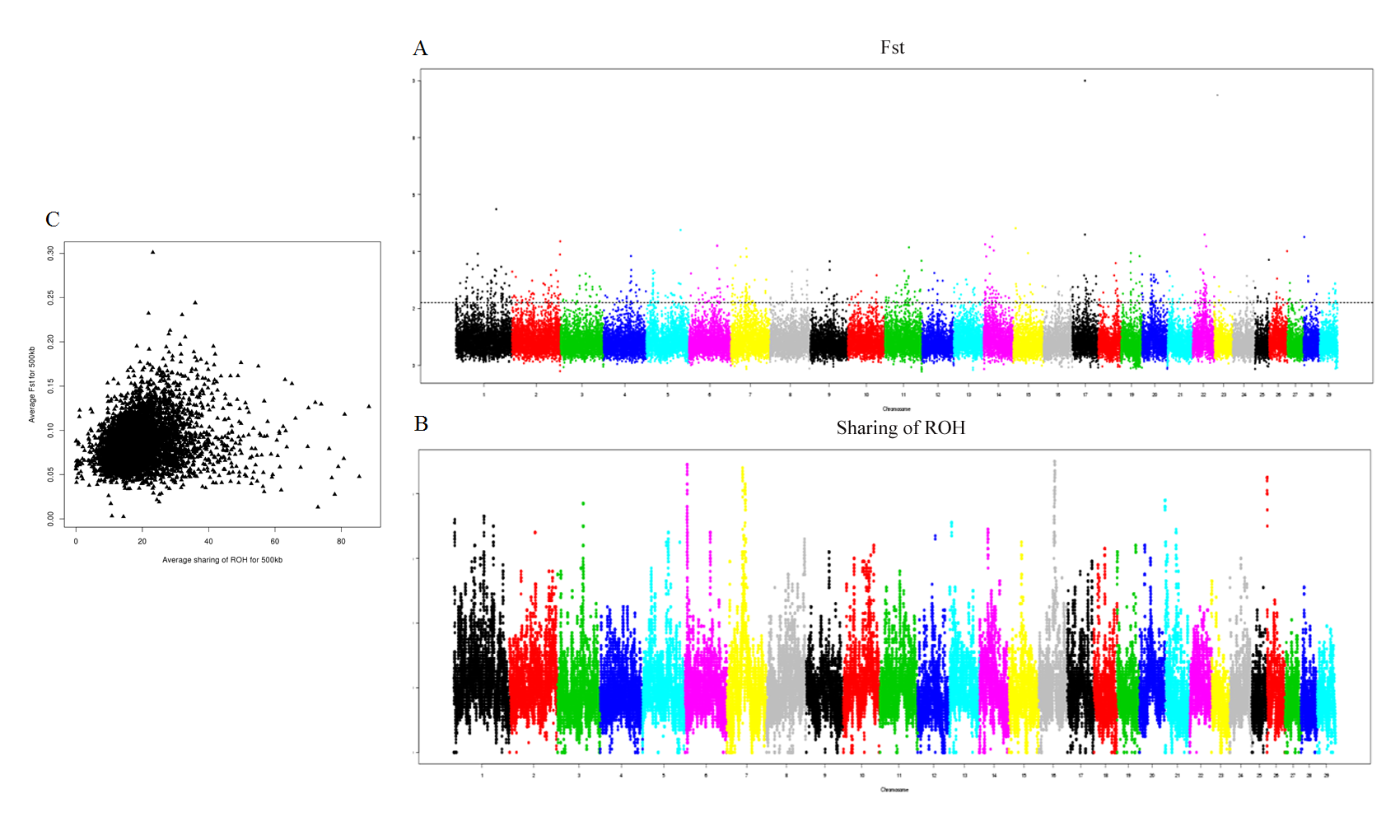

Supplement: Additional file 15: Figure S10. — Signatures of selection based on Fst analysis in sequenced populations. A. Fst values (y-axis) are plotted for each 10 kb bin for all 29 chromosomes. B. The sharing of ROH regions in a 10 kb bin among all individuals for all chromosomes; the y-axis is the number of individuals who have the same region of ROH in a 10 kb bin. C. Correlation between averaged Fst values (y-axis) and number of individuals sharing the same ROH regions in a 500 kb bin (x-axis) (Pearson’s product-moment correlation coefficient = 0.20; p < 0.001). [file 12864_2015_1715_MOESM15_ESM.tiff]

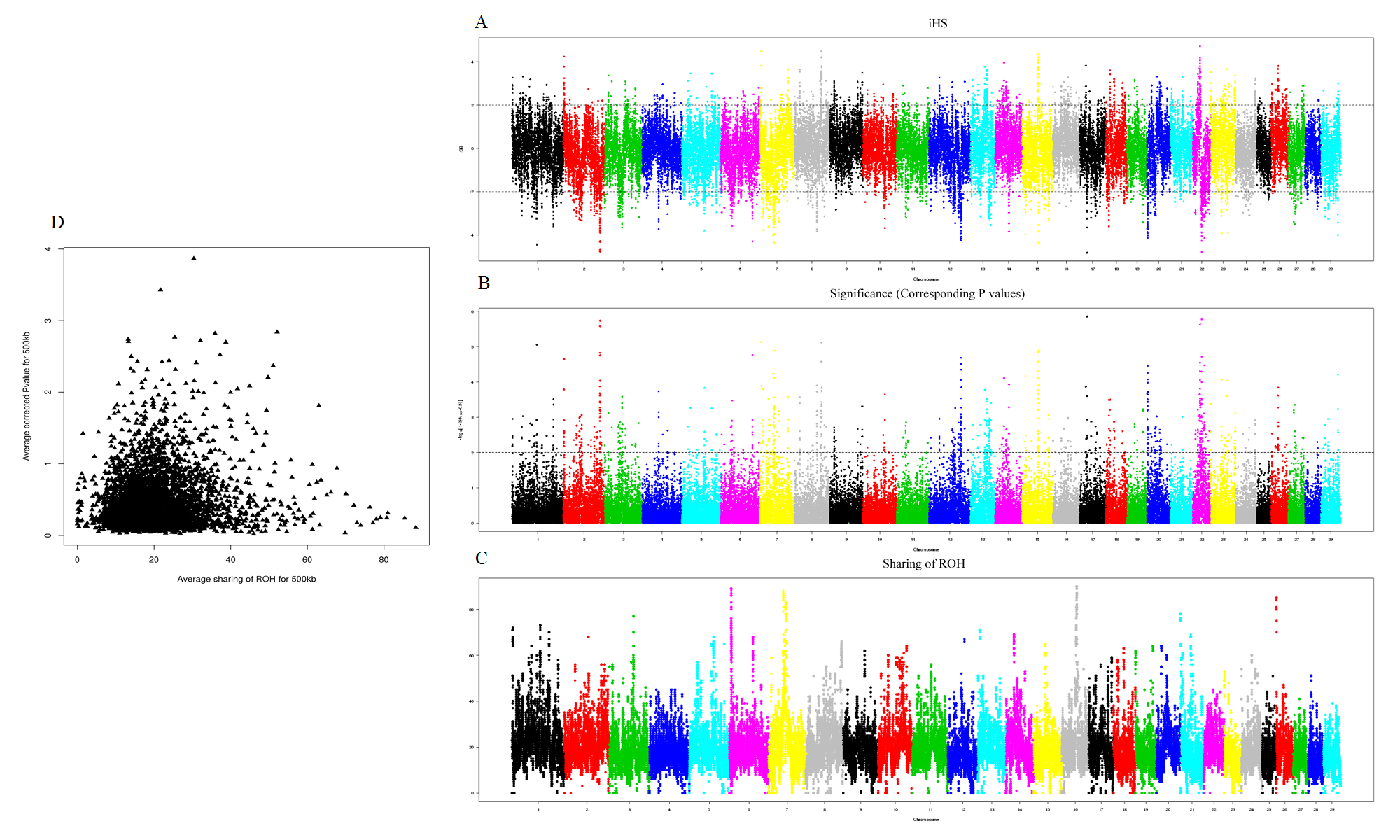

Supplement: Additional file 16: Figure S11. — Signatures of selection based on iHS testing in sequenced populations. A. The iHS signals (y-axis) between sequenced populations are plotted for all 29 chromosomes. B. Significance of the rSB signals (corresponding p values) for sequenced populations for each chromosome. C. The sharing of ROH regions in a 10 kb bin among all individuals for all chromosomes; the y-axis is the number of individuals who have the same ROH region in a 10 kb bin. D. Correlation between p values of iHS signals (y-axis) and number of individuals sharing the same ROH regions in a 500 kb bin (x-axis) (Pearson’s product-moment correlation coefficient = 0.22; p < 0.001). [file 12864_2015_1715_MOESM16_ESM.tiff]

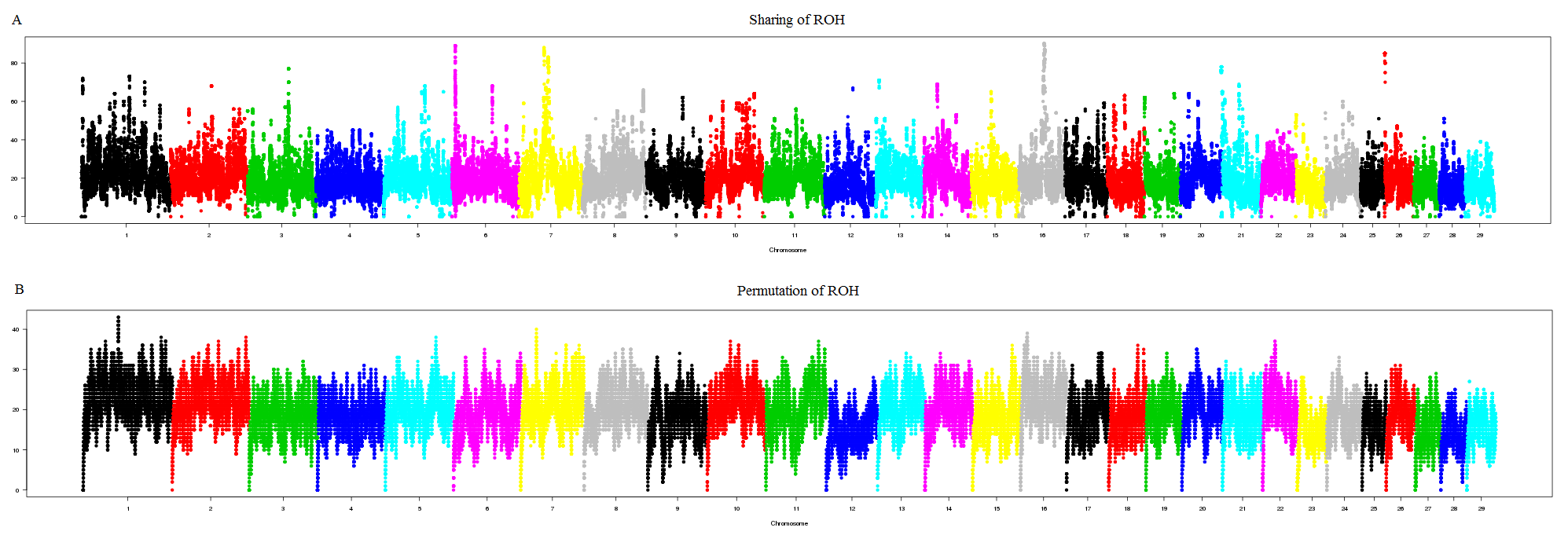

Supplement: Additional file 17: Figure S12. — Permutation of ROH regions compared with sharing of ROH regions among individuals. A. Sharing of ROH regions in a 10 kb bin among all individuals for all chromosomes; the y-axis is the number of individuals who have the same ROH region in a 10 kb bin. B. The randomized ROH regions over genomes in 10 kb bins; the y-axis is the number of individuals who have the same ROH region in a 10 kb bin. [file 12864_2015_1715_MOESM17_ESM.tiff]
